# Optimizing Archaeal Lipid Biosynthesis in *Escherichia coli*

**DOI:** 10.1021/acssynbio.4c00235

**Published:** 2024-08-03

**Authors:** Mirthe Hoekzema, Jiayi Jiang, Arnold J. M. Driessen

**Affiliations:** Department of Molecular Microbiology, Groningen Biomolecular Sciences and Biotechnology Institute, University of Groningen, Nijenborgh 7, 9747AG Groningen, Netherlands

**Keywords:** hybrid membranes, lipid biosynthesis, lipid divide, synthetic isoprenoid utilization pathway, IUP

## Abstract

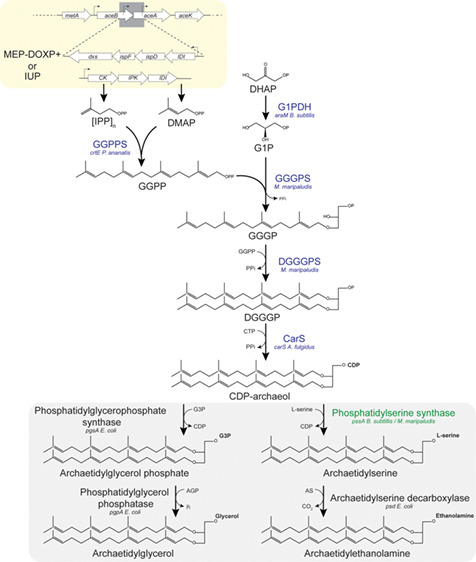

Membrane lipid chemistry is remarkably different in archaea compared with bacteria and eukaryotes. In the evolutionary context, this is also termed the lipid divide and is reflected by distinct biosynthetic pathways. Contemporary organisms have almost without exception only one type of membrane lipid. During early membrane evolution, mixed membrane stages likely occurred, and it was hypothesized that the instability of such mixtures was the driving force for the lipid divide. To examine the compatibility between archaeal and bacterial lipids, the bacterium *Escherichia coli* has been engineered to contain both types of lipids with varying success. Only limited production of archaeal lipid archaetidylethanolamine was achieved. Here, we substantially increased its production in *E. coli* by overexpression of an archaeal phosphatidylserine synthase needed for ethanolamine headgroup attachment. Furthermore, we introduced a synthetic isoprenoid utilization pathway to increase the supply of isopentenyl-diphosphate and dimethylallyl diphosphate. This improved archaeal lipid production substantially. The archaeal phospholipids also served as a substrate for the *E. coli* cardiolipin synthase, resulting in archaeal and novel hybrid archaeal/bacterial cardiolipin species not seen in living organisms before. Growth of the *E. coli* strain with the mixed membrane shows an enhanced sensitivity to the inhibitor of fatty acid biosynthesis, cerulenin, indicating a critical dependence of the engineered *E. coli* strain on its native phospholipids.

## Introduction

All cellular life is surrounded by a membrane, which not only defines the cell as a compartment but also has important cellular functions, such as energy generation, signal transduction, and cell division. Phospholipids are a major component of cellular membranes; they typically form a bilayer, which serves as a barrier for ions and small molecules and forms a matrix in which membrane proteins are embedded. Membrane composition is however remarkably different in archaea compared to bacteria and eukaryotes.^1,2^ While phospholipids in bacteria and eukaryotes typically consist of fatty acids linked to a glycerol-3-phosphate (G3P) via ester bonds, archaeal phospholipids have isoprene-based alkyl chains that are ether-linked to glycerol-1-phosphate (G1P). This difference in chemical structure, known as the “lipid divide”, is reflected by distinct lipid biosynthetic pathways consisting of nonhomologues enzymes in key enzymatic reactions that arose through parallel evolution.^3^ The lipid divide likely occurred during the divergence of bacteria and archaea from the last universal common ancestor and is still visible in the various kingdoms of life as of today. However, the driving force for this split remains unclear.

Over the last decades, the biosynthetic steps leading to archaeal phospholipid biosynthesis have been elucidated.^4,5^ The isoprene units of archaeal phospholipids are synthesized from the isomers isopentenyl pyrophosphate (IPP) and dimethylallyl pyrophosphate (DMAPP). IPP and DMAPP are not solely building blocks for phospholipid biosynthesis but for many different compounds, including steroids, quinones, and carotenoids. Therefore, isoprenoid biosynthetic pathways are found in all three domains of life. Archaea and eukaryotes use the (modified) mevalonate (MVA) pathway for IPP and DMAPP production, while most bacteria use the methylerthritol 4-phosphate (MEP) pathway.^6^ Polyisoprenoid chains of various lengths are formed by the sequential condensation reaction of DMAPP and IPP by enzymes of the isoprenyl pyrophosphate synthase (IPPS) family.^7^ The diverse IPPS enzymes are capable of synthesizing different chain lengths. Most archaeal lipids are composed of C_20_ and/or C_25_ isoprene chains synthesized by geranylgeranyl pyrophosphate (GGPP) synthase and geranylfarnesyl pyrophosphate (FGPP) synthase, respectively.^8,9^ Reduction of dihydroxyacetone phosphate (DHAP) by glycerol-1-phosphate dehydrogenase (G1PDH) produces G1P, the glycerophosphate backbone of archaeal phospholipids. In bacteria, glycerol-3-phosphate dehydrogenase (G3PDH) produces G3P in a similar fashion. However, despite catalyzing a similar reaction, G1P and G3P dehydrogenases share no homology and belong to different protein families and hence are evolutionary not related.^10^ Ether linkage of the first and second isoprenoid chains to G1P is catalyzed by geranylgeranylglyceryl phosphate (GGGP) synthase and di-o-geranylgeranylglyceryl phosphate (DGGGP) synthase, respectively. The resulting DGGGP needs to be converted to CDP-archaeol by CDP-archaeol synthase (CarS) before polar head groups can be attached. The most common headgroups in archaea as well as bacteria are serine, ethanolamine, glycerol, and myo-inositol. The enzymes catalyzing the headgroup modifications are closely related belonging to a superfamily of CDP-alcohol phosphotransferases together with their bacterial and eukaryotic counterparts.^11^ These enzymes are highly promiscuous accepting both bacterial and archaeal substrates.^12^ Finally, the isoprenoid chains of mature archaeal phospholipids are generally fully saturated by geranylgeranyl reductase (GGR).^13^ It is unclear at which step of the biosynthetic process saturation occurs.

It is generally assumed that most contemporary organisms have homochiral membranes, meaning only G3P based lipids in bacteria and eukarya and only G1P based lipids in archaea. Noteworthy exceptions are *Bacillus amyloliquefaciens*, *Bacillus subtilis*, *Clavibacter michiganensis*, and *Geobacillus stearothermophilus* which have a membrane consisting of lipids with fatty acids ester bonded to either G3P or G1P, likely because their genomes contain a glycerol-1-phosphate dehydrogenase (G1PDH) responsible for production of G1P.^14^ Hence, heterochirality does not seem to be a critical event in the lipid divide. Additionally, bacteria of the Fibrobacteres–Chlorobi–Bacteroidetes group superphylum have been reported to encode and express genes involved in the production of typical archaeal ether lipids while also producing typical bacterial phospholipids, potentially leading to membranes with mixed bacterial and archaeal type lipids.^15^ However, the stereochemistry of the produced archaeal lipids remains unclear, meaning this mixed membrane might not be heterochiral.^15−20^ Thus, far, no organism is known to have a mixed heterochiral membrane.

To experimentally probe the compatibility of archaeal and bacterial type lipids, *Escherichia coli* has been engineered to contain such a mixed heterochiral membrane by introducing expression of archaeal lipid biosynthetic genes.^12,21−25^ Lai et al.^21^ and Yokoi et al.^22^ introduced four archaeal lipid biosynthetic genes into *E. coli*, i.e., G1P dehydrogenase, GGPP synthase, GGGP synthase, and DGGGP synthase, from the hyperthermophile, *Archaeoglobus fulgidus*, and the mesophilic methanogen, *Methanosarcina acetivorans*, respectively. To increase the supply of GGPP for ether lipid synthesis, also the native *E. coli* isopentenyl-diphosphate (IPP) isomerase (idi) was overexpressed. This resulted in DGGGP production in the case of the *A. fulgidus* genes, and the DGGGP derivatives digeranylgeranylglycerol (DGGGOH), and digeranylgeranylglyceryl phosphoglycerol (DGGGP-Gro) in the case of the *M. acetivorans* genes. Introducing *M. acetivorans* GGR and ferredoxin in the strain described by Yokoi and co-workers^23^ could fully saturate some of the produced DGGGOH. Despite these engineering efforts, the amounts of ether lipid production were very low making up only 1% of the total phospholipid. Caforio et al.^12^ further expanded the set of enzymes used, critically including CarS from *A. fulgidus*, resulting in archaetidylglycerol (AG) formation. The amount of archaeal lipids produced was substantially improved upon by boosting expression of not only *idi*, but also the other MEP/DOXP pathway genes by insertion of an additional copy on the *E. coli* chromosome, leading to an estimated 20% AG in the membrane.^24^ Recently also yeast was successfully engineered to express archaeal type phospholipids.^26^

Functionality of the native *E. coli* membrane should be considered when engineering its lipid composition. The zwitterionic lipid phosphatidylethanolamine (PE) is a major component of the *E. coli* cellular membrane, accounting for about 75% of total membrane lipids.^27^ Other major membrane lipids in *E. coli* are the anionic phosphatidylglycerol (PG) and cardiolipin (Cl) at roughly 20 and 5% of total membrane lipids, respectively.^27^ Membrane phospholipid composition is important for several cellular functions. PG is required for initiation of DNA replication, and protein translocation, while PE has been shown to support sugar and amino acid transport systems as well as aid in membrane protein folding.^28^ PG and PE are both essential phospholipids of the *E. coli* membrane. Although the PG requirement is due to its anionic character, under specific conditions, it can be replaced for other anionic phospholipids, such as phosphatidyl acid (PA) or phosphatidylserine (PS), while unsaturated PE is critical for membrane function due to its polymorphic character, i.e., formation of nonbilayer structures.^28^ To replace the native phospholipid composition of *E. coli* for archaeal phospholipid, production of the corresponding archaeal lipids archaetidylethanolamine (AE) and archaetidylglycerol (AG) is thus preferred to at least maintain the polar headgroup composition. Although it is not known if unsaturated AE exhibits a similar polymorphic behavior as PE.

Here, we describe the continued engineering of a hybrid heterochiral *E. coli* strain^12^ by introducing AE synthesis and a synthetic isoprenoid utilization pathway (IUP), expanding the pallet of archaeal lipids produced also to archaetidylethanolamine and improving the isoprenoid precursor production.

## Results and Discussion

### Introducing Archaetidylethanolamine Formation in *E. coli*

**Figure 1:**
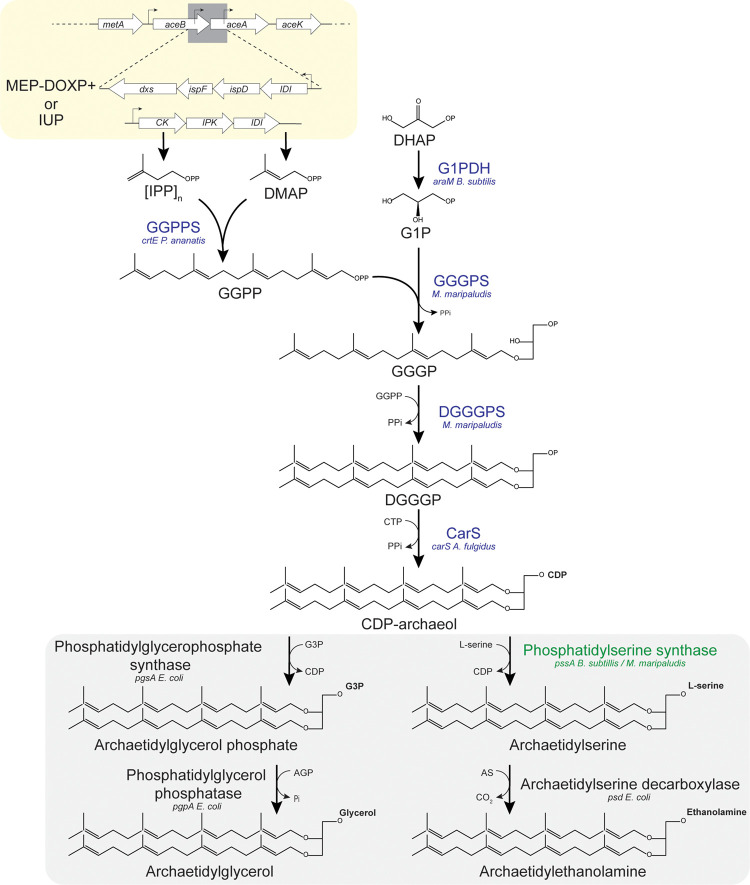
Schematic representation of ether lipid biosynthetic pathway introduced into *E. coli*. Overproduction of isoprenoid precursors DMAPP and IPP by insertion of an additional copy of MEP/DOXP pathway genes *DXS*, *ispD*, *ispF*, and *IDI* onto the chromosome. Alternatively, a synthetic isoprenoid utilization pathway (IUP) consisting of a promiscuous kinase, IPK, and IDI is integrated on the chromosome or supplied on a plasmid. EL biosynthesis genes (blue) are provided on two compatible vectors. For polar headgroup attachment, the native *E. coli* enzymes are utilized (gray box) except for phosphatidylserine synthase (green), an additional heterologous copy is provided when indicated.

Until now, only small amounts of AE production were observed in the engineered *E. coli* strains.^24^ Here, we aim to introduce AE production in our previously described engineered *E. coli* strain^24^ that contains a composite pathway of both bacterial and archaeal enzymes for ether lipid production and upregulates the MEP/DOXP operon responsible for IPP and DMAPP synthesis to achieve higher ether lipid yields (Figure 1). As mentioned before, headgroup modifying enzymes are highly promiscuous accepting both the archaeal and bacterial substrate. PE and AE are synthesized from respectively CDP-diacylglycerol (CDP-DAG) and CDP-archaeol, in two steps; First, phosphatidylserine synthase (Pss) exchanges CDP for l-serine forming phosphatidylserine (PS) or archaetidyl serine (AS), subsequent decarboxylation of PS/AS by phosphatidylserine decarboxylase (Psd) produces PE/AE.^27,28^ However, *E. coli* type I PssA (EcPssA) appears to distinguish between the bacterial and archaeal substrates and appears not as active with CDP-archaeol as compared to CDP-DAG.^29^ Furthermore, purified EcPssA did not show any activity against CDP-Archaeol in vitro.^12^ Our previous attempt to introduce AE production into *E. coli* therefore introduced *B. subtilis* type II PssA (BsPssA) together with the archaeal lipid biosynthetic genes. A crude cell lysate of *B. subtilis* could form AE when supplied with archaeal substrates.^29^ In vitro assays with purified *B. subtilis* PssA (BsPssA) also show activity toward both CDP-DAG and CDP-archaeol^12^ However, this did not lead to a substantial production of AE when expressed in *E. coli*.^24^

**Figure 2:**
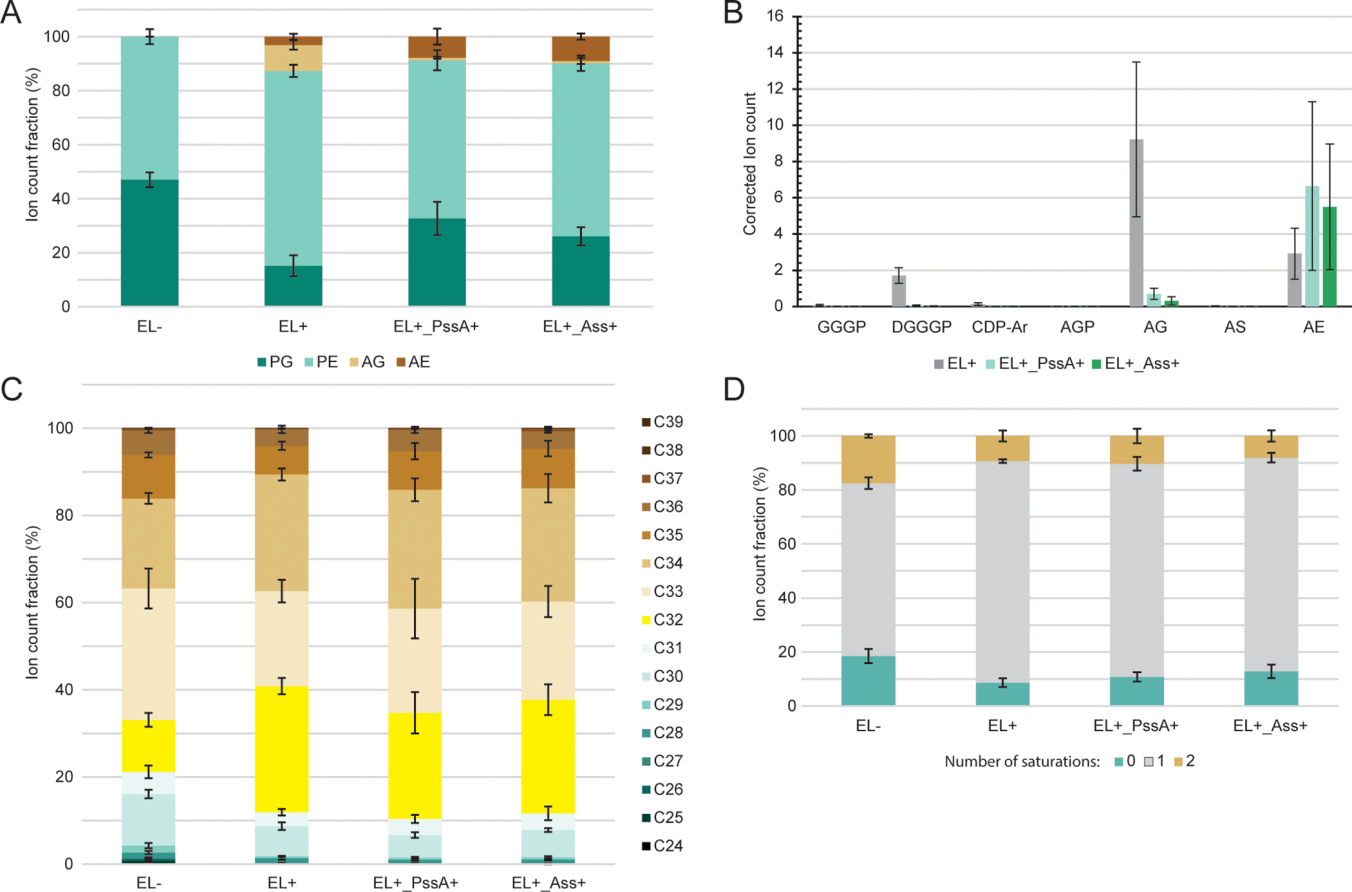
Quantification of in vivo archaeal lipid synthesis in engineered *E. coli* strains. LC-MS analysis of lipid extracts from strains without (EL−) and with (EL+) archaeal ether lipid biosynthesis pathway, including PssA overexpression (EL+_PssA+) and Ass overexpression (EL+_Ass+) grown for 16 h in LB with 10 μM IPTG for induction of EL biosynthesis genes. (A) Breakdown of native phospholipids PG and PE as well as introduced lipids AG and AE. Combined ion counts of all quantified PG, PE, AG, and AE species is 100%). (B) Lipid species AG and AE as well as their precursors GGGP, DGGGP, CDP-archaeol, AGP, and AS, normalized for internal standard. (C) Changes in PG and PE acyl-chain length distribution upon archaeal lipid expression. 100% = all PG and PE species quantified. (D) Same but for the number of saturations in the acyl-chains. Data reflect averages ± SD (*n* = 5 except EL– there *N* = 3).

**Figure 3:**
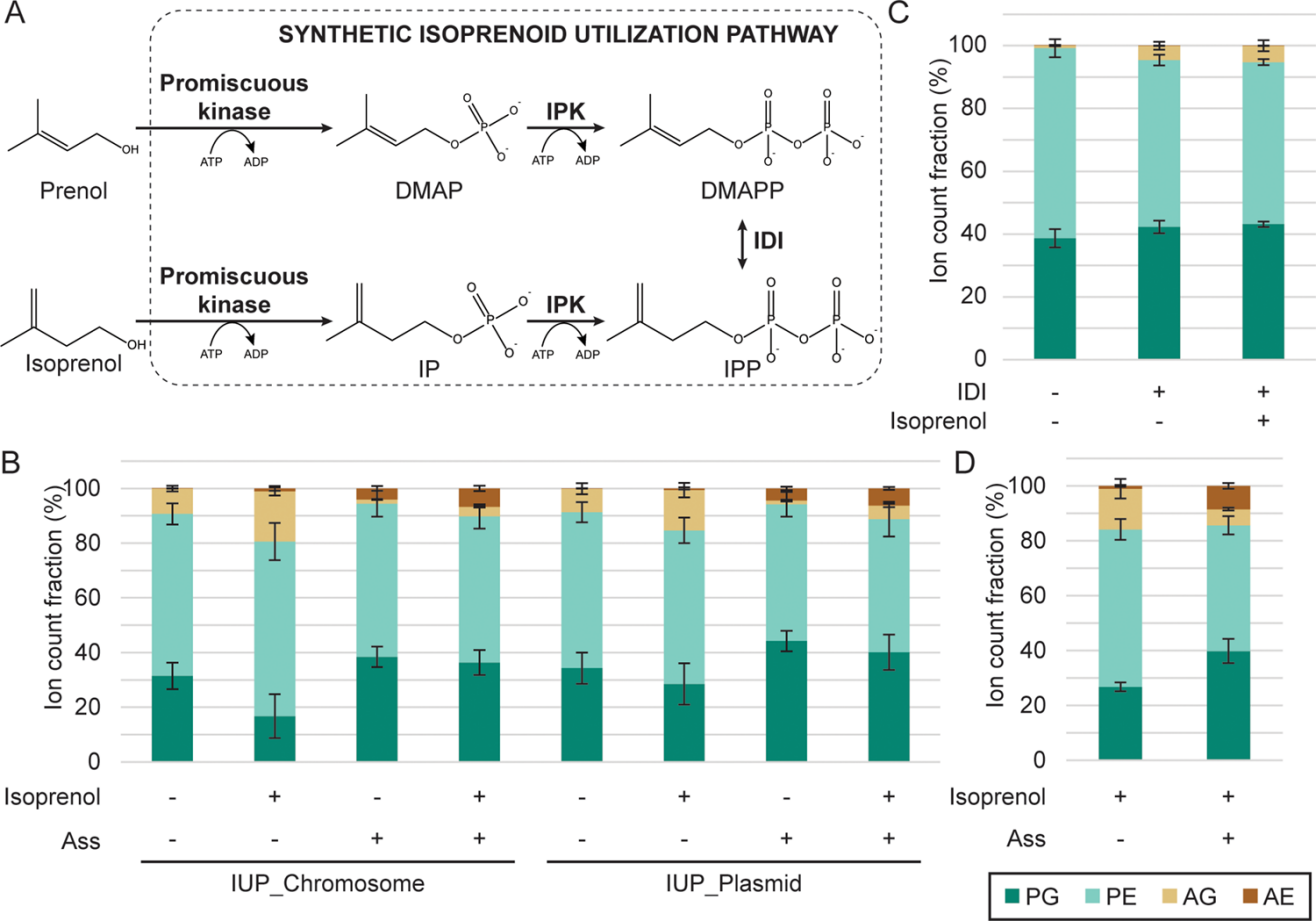
Introduction of a synthetic isoprenoid utilization pathway (IUP) leads to increased archaeal lipid production. (A) IUPs consist of a promiscuous kinase and isopentenyl phosphate kinase (IPK) which catalyze the reactions to convert externally supplied prenol or isoprenol into the isoprenoid building blocks DMAPP and IPP. Isopentenyl-diphosphate delta-isomerase (IDI), which interconverts DMAPP and IPP, is included to ensure a balanced ratio between DMAPP and IPP. (B) LC-MS analysis of lipid extracts from strains with the archaeal lipid biosynthetic pathway ± Ass overexpression and the IUP (ScCK, AtIPK, EcIDI) either integrated on the chromosome or provided on a plasmid, grown for 16 h in M9 with 10 μM IPTG for induction of EL biosynthesis genes, when indicated 25 mM isoprenol was added to the media. Shown is the ratio between PG, PE, AG, and AE lipid species, where 100% is the total ion count of all species quantified. (C) LC-MS analysis of lipid extracts from strains with the archaeal lipid biosynthetic pathway with and without overexpression of IDI from a plasmid grown for 16 h in M9 with 10 μM IPTG in the presence and absence of 25 mM isoprenol. Ratios of PG, PE, AG, and AE are shown. (D) LC-MS analysis of lipid extracts from strains with the archaeal lipid biosynthetic pathway and IUP version 2 (SmDAGK, MvIPK, EcIDI) from a plasmid grown for 16 h in M9 with 10 μM IPTG in the presence of 25 mM isoprenol. Ratios of PG, PE, AG, and AE are shown as before. Data reflect averages ± SD (*N* = 3).

After inspection of the vectors expressing the archaeal lipid biosynthetic genes and BsPssA, some potential problems with BsPssA expression were identified. Vector pAC09 expressed *carS*, *gggps*, *dgggps*, and *B. subtilis* pssA in that order from one IPTG inducible T7 promoter. Hence, the expression of BsPssA might be limited, as it was furthest away from the promoter. Furthermore, two start codons were detected between the RBS and *carS* open reading frame, one out of frame leading to a nonsense product thereby potentially reducing CarS expression (Figure S1). Hence, we deleted the out of frame start codon in front of *carS*, and cloned *B. subtilis* pssA behind a second IPTG inducible T7 promoter to boost expression levels. Instead of bacterial we also cloned a *pssA* from archaeal origin, specifically *Methanococcus maripaludis*, to test if this would increase AE production. PssA genes from archaeal origin are sometimes called archaetidylserine synthase (*ass*), a designation we keep throughout this paper to distinguish between PssA from bacterial and archaeal origin. We also included a strain without any Pss or Ass overexpression; in this case, only native *E. coli* PssA is present at native levels. Strains were grown for 16 h in LB while ether lipid biosynthetic genes were minimally induced with 10 μM IPTG. Lipids were extracted from lyophilized cells using an adapted acidic Bligh and Dyer method employing 0.1 M HCl.^30,31^ Lipid species were analyzed by liquid chromatography–mass spectrometry (LC-MS), using a RP-UHPLC-ESI-MS method designed to separate complex biological lipid mixtures.^32^ The resulting lipidome was subsequently analyzed for the presence of AE, AG and their precursors, as well as 33 PG and 40 PE native *E. coli* phospholipid species that were reported before as well separated by the LC method used^32^ (Tables S1–S3). Based on the obtained ion counts, an approximation of lipid ratios was determined. Overexpression of BsPssA or MmAss results in AE production at, respectively, 8 and 9% of total phospholipids quantified (Figure 2A,B). However, concomitantly, the AG production decreased 10-fold (Figure 2A). This is likely a consequence of a competition between the AE and AG pathways for the common precursor CDP-archaeol which is detected only in very low amounts (Figure 3 B). Interestingly we do observe production of AE in the strain with only native EcPssA, although at a lower level (3%) (Figure 2A,B). This clearly shows that EcPssA does accept CDP-Archaeol as a substrate in vivo, in contrast to previous in vitro assays.^12,29^ It has been reported that the activity of *E. coli* PssA depends on the presence of anionic lipids in the matrix it is operating in.^33,34^ The in vitro assay with isolated EcPssA was performed in detergent, and this sub-optimal environment could explain the lack of activity toward the archaeal substrate.^12^ This theory is substantiated by the very low amounts of PS detected in the presence of the natural substrate PA.^12^ The study using an *E. coli* membrane fraction does not suffer from this problem, and while a decrease in activity was observed with the archaeal substrate compared to the bacterial substrate, some activity remained.^29^ It is not inconceivable that substrate availability would play a role in this, as PA would be present in the membrane fraction itself, while it is uncertain where the added archaeal substrate localizes.

Since the polymorphic character of PE fulfills an important role in the *E. coli* membrane, we also determined the acyl-chain composition of the bacterial phospholipid pool. For that reason, ion counts of all lipids with the same tail length (Figure 2C) or number of saturations (Figure 2D) were combined and expressed as % of the total bacterial phospholipid ion count (33 PG, 40 PE species). The average tail length remains essentially identical with overexpression of ether lipids; 32.8 without EL expression, to 32.9 with EL expression, and 33.1 and 33.0% when EL expression is combined with, respectively, PssA and Ass expression, respectively. We observe, however, more monosaturated lipids when ether lipids are expressed, around 80% with EL versus 64% without (Figure 2D). This is accompanied by reduced numbers of both di- as well as unsaturated phospholipids.

### Introduction of a Synthetic IUP

In the engineered EL-pathway *E. coli* strain, we observe no accumulation of archaeal lipid precursors (Figure 2B). Further, as discussed above, AE and AG production appears to be limited by the availability of CDP-archaeol. Overall, this suggests that the limiting factor for EL production occurs before GGPP formation (Figure 2B). Upregulation of DMAPP and IPP production has previously been reported to substantially increase EL formation^24^ and might thus be a good target for further improvement. DMAPP and IPP are the precursors for isoprenoids, a large family of natural products that includes the isoprene chains used in EL biosynthesis. In *E. coli* DMAPP and IPP are produced by the MEP/DOXP operon. The original EL *E. coli* strain has an additional copy of this operon inserted into the chromosome (Figure 1). However, the MEP/DOXP pathway is coupled to the central carbon metabolism and is therefore tightly regulated, limiting DMAPP and IPP production. Recently a synthetic IUP was developed.^35^ In this two-step pathway, IPP and DMAPP are synthesized from externally provided prenol or isoprenol in two consecutive phosphorylation steps, catalyzed by a promiscuous choline kinase from *Saccharomyces cerevisiae* (ScCK) and isopentenyl phosphate kinase (IPK) from *Arabidopsis thaliana* (AtIPK), respectively (Figure 3A). To balance the ratio of DMAPP and IPP, *E. coli* isopentenyl-diphosphate delta-isomerase (IDI) was included. This IUP thus decouples DMAPP and IPP production from central carbon metabolism and regulation, thereby being able to sustain a high metabolic flux toward isoprenoid production.^35^ We transferred the IUP described by Chatzivasileiou et al.^35^ into the *E. coli* BL21 chromosome at the Ori macrodomain or to a compatible vector and added the ether lipid expression vectors ± MmAss overexpression to these strains. Cells were grown for 16 h in M9 with 0.4% glucose and 0.5% casamino acid instead of LB to avoid ScCK to act on choline which is present in the yeast extract added to LB. Expression of ether lipid biosynthesis genes was induced with 10 μM IPTG and when indicated 25 mM isoprenol was supplied in the media for DMAPP and IPP production. This concentration of isoprenol was chosen because it was reported to be the optimal condition for lycopene production using the same IUP.^35^ Lipids were extracted and analyzed by LC-MS (Tables S4–S6). When both the IUP and isoprenol are present we observe a marked increase in ether lipid production, from 10% AG 3% AE to 18% AG 1% AE with chromosomal and 15% AG 1% AE with plasmid-based IUP expression (Figure 2A,B). This indicates that the DMAPP and IPP supply was indeed a limiting factor for EL synthesis. For the strain carrying the Ass overexpression vector, AG production goes up from 1% to 4% and 5% with the IUP, however, AE goes down from 9% to 7% and 6%, making the total amount of ether lipids produces comparable. It is unclear why the IUP enhanced only AG production. Surprisingly, when no isoprenol is supplied, we still observe EL production at around half of that observed in the presence of isoprenol. As IDI is a key rate-limiting step in isoprenoid production and overexpression of different IDIs in *E. coli* has been reported to increase isoprenoid yields,^36^ we suspected that the remaining ether lipid production could be attributed to IDI overexpression alone. Indeed, when we remove CK and IPK from the IUP cassette, leaving only IDI, an amount of EL production is observed similar to that in the strain with the entire IUP in the absence of isoprenol (Figure 3C). Moreover, this stain is insensitive to isoprenol addition (Figure 3C, compare lanes 2, 3). Importantly, a strain without MEP/DOXP, IUP, or IDI overexpression produces minimal amounts of ether lipids (0.8%), meaning increased EL yields are related to IUP or IDI-only overexpression and not a result of baseline *E. coli* DMAPP and IPP production (Figure 3C, compare lanes 1, 2).

The marked increase of ether lipid production after the introduction of the IUP is not accompanied by an increase in precursors (Figure S2). Hence, the supply of DMAPP or IPP is likely still a limiting factor. Different versions of the IUP were developed to further optimize isoprenoid production in *E. coli*.^37^ A side-by-side comparison of the different enzyme variants used to build these IUPs revealed that diacylglycerol kinase (DAGK) from *Streptococcus mutans* (SmDAGK) and an IPK from *Methanococcus vannielii* (MvIPK) was the most optimal enzyme combination.^37^ We therefore replaced ScCK with SmDAGK and AtIPK with MvIPK on our IUP expression vector (IUP-V2). Relative quantitation of the phospholipids detected by LC-MS indicate that there is no marked increase in EL production with the IUP-V2 (Figure 3B, lanes 6 and 8, Figure 3D). This is somewhat surprising since Ma and co-workers reported that under all the conditions tested SmDAGK performed better than ScCK in lycopene and isoprenoid production.^37^

If other factors are limiting EL production, this would obscure any positive effect of the different enzyme variants on EL production. For example, while we use the original constitutive pro4 promoter, Ma and co-workers use the inducible pBad promoter,^35^ it is possible that with the optimized inducer concentrations found by Ma et al, the pBad promoter outperforms the pro4 promoter.^35^ Another possibility is a bottleneck at the conversion of DMAPP and IPP to GGPP by CrtE. It has been reported that, after successful DMAPP and IPP overexpression, GGPP formation is the rate-limiting step in carotenoid biosynthesis.^38^ Since we cannot determine GGPP concentrations with our LC-MS method, we cannot exclude that GGPP formation is a limiting factor. Finally, we cannot rule out whether we have reached the limit of archaeal lipid incorporation into the *E. coli* membrane.

Summarizing, the use of a synthetic IUP enhances archaeal lipid expression in engineered *E. coli* strains. This effect can in part be attributed to IDI overexpression, but with the addition of isoprenol to the media the IUP produces three times as much EL as with just IDI.

### Production of Hybrid Cardiolipin Species

**Figure 4:**
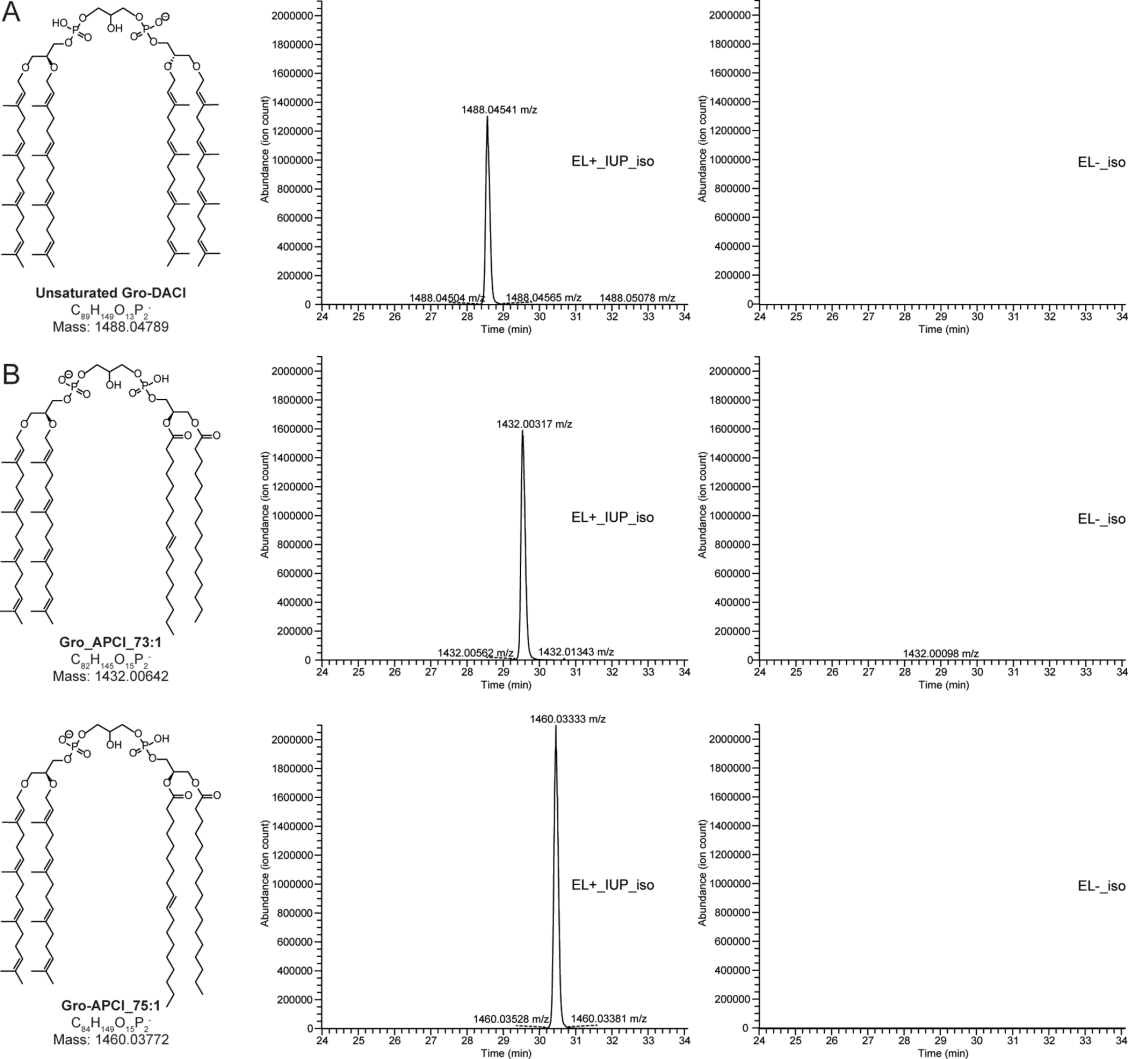
In vivo synthesis of bacterial-archaeal hybrid cardiolipin species. (A) Structure of unsaturated Gro-DACL and LC-MS chromatogram of a strain with (middle panel) and without (right panel) ether lipid expression, filtered for the mass of Gro-DACL (mass tolerance of 5 ppm). (B) Structure of the two most abundant Gro-APCL species and LC-MS chromatograms (filtered for mass, tolerance 5 ppm), showing lipid species can be detected in the lipidome of a strain with ether lipid expression, but not in a strain without.

A third major component of the *E. coli* membrane is cardiolipin^27^ that in stationary phase cells can amount to 5% of the overall phospholipid.^27,39,40^ Cardiolipin (Cl) consists of two phospholipids bridged by a polar headgroup, typically glycerol-diphosphatidyl-cardiolipin (Gro-DPCL). We searched the lipidomes of our IUP engineered strains for the hypothetical masses of cardiolipins based on the incorporation of one or two AG molecules. Indeed, the lipidome contains the archaeal cardiolipin (glycerol-diarchaetidyl-cardiolipin or Gro-DACL) as well as bacterial-archaeal hybrid cardiolipin species (glycerol-archaetidyl-phosphatidyl-cardiolipin, or Gro-APCL) of which 16 variants were found differing in the acyl-chain composition of the PG moiety (Figure 4A,B and Table S7). Such variants were previously reported in vitro using the Cls of the methanogenic archaeon *Methanospirillum hungatei* (MhCls)^41^ that was purified from an overexpressing *E. coli* strain but have never been observed in vivo. The cardiolipins (Gro-DPCL, Gro-DACL, and Gro-APCL species) constitute on average 2% of total phospholipids quantified (CL, PG, PE, AG, and AE species). The distribution of bacterial, archaeal, and hybrid cardiolipin species varies (Figure S3). As expected, there is a slight increase in the ratio of Gro-DACL to Gro-APCl when isoprenol is added to the media, reflecting the increase in AG content. In strains with MmAss overexpression, the ratio of archaeal and hybrid Cl species is substantially lower than in those without, which might be a reflection of the decreased amounts of AG, which is a potential substrate for the endogenous cardiolipin synthase.

In conclusion, one or more *E. coli* cardiolipin synthases accept AG as a substrate creating hybrid cardiolipin species in vivo. As cardiolipin species constitute only a minor part of the phospholipids present in our engineered strain under the conditions tested, this does not substantially change previously reported ratios of ether lipids.

### Archaeal Lipid Production under FAS Inhibition

**Figure 5:**
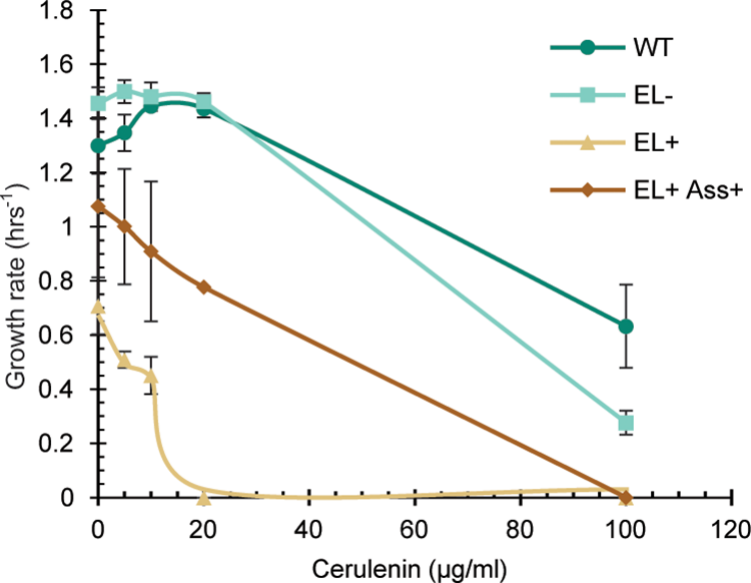
Growth rates of cerulenin treated cultures. Optical density of engineered cultures incubated with varying concentrations of the FAS inhibitor cerulenin was monitored in a plate reader. Growth rates were determined using the Growthcurver R-package and averaged and SD were plotted (*N* = 3).

Phospholipid production is tightly regulated in *E. coli* to synchronize with cellular growth and division.^42,43^ In our engineered strain, overexpression of the EL-pathway enzymes results in the continuous synthesis of ether lipids. Indeed, strong induction of ether lipid expression (100 μM IPTG and higher) resulted in cell morphology changes, and the shredding of vesicles from the membrane surface with the same mixed lipid composition as the cytoplasmic membrane.^24^ Further, a high level of expression caused nonsymmetrical cell division and growth defects. Importantly, these morphological changes do not occur when low expression levels of the ether lipid genes are induced (at 10 μM IPTG or lower). A potential approach to improve ether lipid production in *E. coli* is to limit the production of the native phospholipids, but this would only work when *E. coli* does not differentiate between the endogenous and archaeal lipids for functioning. A key enzyme complex in phospholipid biosynthesis is the type II fatty acid synthase (FASII)^44^ that is responsible for the formation of acyl-CoA. FASII is an essential enzyme complex and has been a major target for antibiotics development.^44,45^ The antibiotic cerulenin inhibits the condensation of acyl- and malonyl-ACP by FabB/FabF, thereby blocking the elongation of the acyl chains.^46^ When cerulenin is added to *E. coli*, growth is inhibited in a dose-dependent fashion, i.e., at 100 μg/mL, the growth rate of *E. coli* is reduced by 40%.^47^ To determine if ether lipid biosynthesis, which is independent of FASII, could negate growth inhibition by cerulenin, growth of our engineered strains (MEP/DOXP+, EL+, and EL+Ass) was tested in the presence of 0, 5, 10, 20, and 100 μg/mL cerulenin using a plate reader. The MEP/DOXP+ strain without EL expression (EL−) was included as a control as well as the parental strain *E. coli* JM109 (wt). The effect of 5, 10, and 20 μg/mL cerulenin on the growth rate of the wt and EL– strain is negligible, but at a concentration of 100 μg/mL, the growth rate was reduced by 50 and 80%, respectively (Figure 5). As reported previously, in the absence of cerulenin, the strain with ether lipid production showed a lower growth rate compared to the control strains.^24^ Remarkably, strains with EL expression showed increased sensitivity to cerulenin inhibition. A transcriptional profiling study of *E. coli* challenged with 37 antibiotics, showed 627 differentially regulated genes upon treatment with cerulenin, most notably an upregulation of the biotin biosynthetic cluster, which occurred upon treatment with all FAS inhibitors tested.^48^ Cerulenin treatment thus has a broader cellular effect, which could have a negative effect in combination with ether lipid expression. We occasionally observed sudden growth for the EL+ and EL+ PssA+ strains treated with cerulenin after prolonged incubation (Figure S4). When diluted in fresh media with cerulenin, these cells continued to grow (data not shown). A single point mutation in FabF (I108F) can confer resistance to cerulenin,^49^ however, we did not observe this specific mutation in the escape mutants tested. Our data suggest that the engineered strains are critically dependent on endogenous phospholipid biosynthesis, which poses further challenges for increasing the ratio of ether lipids in the membrane.

## Materials and Methods

### Growth Conditions

For all cloning purposes, *E. coli* strains were grown under aerobic conditions in LB-medium (Lennox, Roth) at 37 °C. For growth on plates, 1.5% agar (BD) was added. Antibiotics were included where appropriate; ampicillin 100 μg/mL, kanamycin 50 μg/mL, chloramphenicol 30 μg/mL, and tetracycline 15 μg/mL.

Engineered MEP/DOXP+ strains were grown in 100 mL of LB, with antibiotic selection and 10 μM IPTG. Cultures were incubated in 500 mL flasks placed in a rotary incubator set to 37 °C and 200 rpm for 16 h. IUP engineered strains were grown in 100 mL M9 (1× M9 minimal salts (BD), 2 mM MgSO_4_, 0.1 mM CaCl_2_) with 0.4% glucose, 0.5% casamino acids (Gibco), antibiotic selection, and 10 μM IPTG, when indicated 25 mM isoprenol (Sigma-Aldrich) was added. To contain the volatile isoprenol, all cultures were incubated in 500 mL serum bottles and placed in a 37 °C water bath on top of a magnetic stirring plate set to 400 rpm for 16 h.

### Strains and Cloning Procedures

All strains used are derivatives from *E. coli* K12 JM109(DE3) or *E. coli* B BL21(DE3), and *E. coli* DH5α was used for cloning purposes. Strains and plasmids are listed in Supplementary Tables S8 and S9, respectively. Sequences of primers used can be found in Table S10. The enzymes of the archaeal lipid biosynthetic pathway (Figure 1 and Table S11) were introduced into the engineered strains on two compatible vectors. G1PDH (*B. subtilis* araM) and GGPPS (*Pantoea ananatis crtE*) are provided on pMS148, described in.^24^ CarS (*Archaeoglobus fulgidus*), GGGPS and DGGGPS (both from *M. maripaludis*) are supplied on a second vector pMH09. To create this vector GGGPS and DGGGPS were amplified from pAC027^24^ using primers MHO-022/023 and cloned into pRSF-Duet using *Eco*RI and SacI restriction sites. The resulting vector was linearized with primers MHO-024/025 and recircularized, this introduced a silent point mutation eliminating a NdeI restriction site and removing the N-terminal 6×His tag from DGGGPS. Finally, *carS* amplified from pAC027 with MHO-021/026, was added using NcoI and *Bam*HI restriction sites, resulting in pMH09. *B. subtilis* pssA was amplified from pAC029 using primers MHO-027/028 and cloned after its own P_T7lac_ in pMH09 using NdeI and PvuI restriction sites, resulting in pMH10. A codon-optimized synthetic gene of *M. maripaludisass* was ordered from GeneArt (ThermoFisher Scientific), amplified with primers MHO-031/032, and added to pMH09 in a similar fashion creating pMH12.

For increased production of isoprenoids, the previously described MEP/DOXP+ strain was used unless indicated otherwise.^24^ Alternatively, strains were used with the synthetic IUP integrated into the chromosome of *E. coli* BL21(DE3) or supplied on separate compatible plasmid. Genes used for both pathways are given in Table S12. For scarless integration of the IUP on the *E. coli* chromosome, a *cat-sacB* counter selectable cassette (GenBank KM018298) was amplified using primers MHO-071/072 introducing homologue ends. It was integrated onto the *E. coli* BL21(DE3) chromosome in the Ori macrodomain using Lambda Red mediated recombination,^50^ selecting for chloramphenicol resistance. Next, the entire IUP (P_pro4__*ck*, *ipk*, *idi*) was amplified from pSEVA228-pro4IUPi (AddGene Plasmid #122018),^35^ using primers MHO-073/074 introducing the same homologue ends. The PCR product was used in a second recombination step to replace the *cat-sacB* cassette. By plating transformants on LB plates with 5% sucrose, cells with successful integration, thereby lacking toxic SacB expression, were selected. For plasmid-based expression, the IUP was amplified from pSEVA228-pro4IUPi using primers MHO-077/078 introducing *Bam*HI and NotI restriction sites. From pACYC-Duet, the P15A origin of replication, chloramphenicol resistance marker, and P_T7lac_ promoter were amplified with primers MHO-079/080 also introducing *Bam*HI, and NotI restriction sites. PCR products were digested and ligated to create the IUP expression vector pMH27. To create the IDI-only expression vector pMH35, the P_T7lac_ promoter, P15A origin, chloramphenicol marker, and idi were amplified from pMH27 using primers MHO-090/091 and circularized. For IUP-V2 codon-optimized synthetic genes of *S. mutans* dagk and *M. vannieliiipk* were ordered from GeneArt (ThermoFisher Scientific) and amplified with primers MHO-123/124 and MHO-125/126, respectively. The P_T7lac_ promoter, P15A origin, chloramphenicol marker, and idi were amplified from pMH27 with primers MHO-127/128. All PCR fragments were assembled using Gibson assembly mix (NEB) according to protocol, creating IUP-V2 expression vector pMH42.

### Total Lipid Extraction

For lipid extraction strains were grown as detailed above, after which cells were harvested by centrifugation (3500 *g*, 10 min at 4 °C). Cell pellets were snap frozen in liquid nitrogen and subsequently lyophilized for 24 h. Lipids were extracted from 10 mg freeze-dried cells using an adapted acidic Bligh and Dyer method using 0.1 M HCl instead of 2 M HCl or 5% trichloroacetic acid as described elsewhere.^13,30^ As internal standard, 10 μg of dodecyl-β-D-maltoside (DDM) was added before extraction. The crude chloroform fraction was dried under a nitrogen stream, and the resulting lipid film was re-extracted three times with 400 μL chloroform. This process was repeated with chloroform: methanol (1:2) and methanol. The final lipid film was resuspended in methanol to a concentration of approximately 5 mg/mL before LC-MS analysis.

### LC-MS Analysis of Lipids

Total lipid extracts were analyzed using an Accela1250 UHPLC system (Thermo Fisher Scientific) coupled with a Thermo Exactive Orbitrap mass spectrometer (Thermo Fisher Scientific) equipped with an ESI ion source. A sample of 5 μl was injected into the ACQUITY Premier CSH C18 (1.7 μM 2.1 × 150 mm) column (Waters Chromatography Ireland). Separation of lipids was achieved using a gradient of Milli-Q-H_2_O:MeCN (40:60) and Milli-Q-H_2_O:MeCN:1-BuOH (0.5:10:90) both with 5 mM ammonium formate as described.^32^ The column effluent was injected directly into the Exactive ESI-MS Orbitrap operating in negative ion mode. Voltage parameters: spray 3.0 kV, capillary −75 V, tube lens −190 V, skimmer −46 V. Capillary temperature set to 300 °C, sheath gas and auxiliary gas flow 60 and 5 units, respectively. Spectral data were analyzed using the Thermo Scientific Xcalibur processing software. Ion counts were obtained by applying the Genesis algorithm based on automated peak area detection and integrations. Peaks were checked manually to make sure the right lipid species was detected. Total ion counts of ether lipid species (Figure 2B) were normalized for DDM (*m*/*z* 509.3 [M – H]^−^) and plotted as normalized ion counts. For ratios of archaeal lipids versus bacterial lipids (Figures 2A and 3B–D), the combined ion counts of all quantified lipid species (but not precursors) were assumed to be 100%. A similar approach was used to obtain ratios for chain length and saturation status of all bacterial phospholipids quantified (Figure 2C,D); however, ether lipids were excluded.

### Growth Experiments FAS Inhibition

Overnight cultures were diluted 1:100 in M9 (1× M9 minimal salts, 2 mM MgSO_4_, 0.1 mM CaCl_2_) with 0.4% glucose, 0.5% casamino acid, ampicillin (100 μg/mL), kanamycin (50 μg/mL), and IPTG (10 μM). The diluted overnight cultures were divided over Eppendorf tubes, and 0, 5, 10, 20, and 100 ug/mL cerulenin of *Cephalosporium caerulens* (Merck, 219557) was added. From each of the diluted overnight cultures with cerulenin, 200 μL was immediately added to a 96-well cell culture plate (Sarstedt, ref 83.3925) and covered with a gas permeable sealing membrane for microtiter plates (Breath-Easy, DiversifieDBiotech). The plate was incubated in a PowerWave 340 plate reader (BioTek) at 37 °C with continuous fast shaking, and OD639 was measured every 10 min. Growth rates were determined using the Growthcurver R-package.^51^

## Supporting Information Available

Schematic of the ether lipid biosynthesis pathway introduced into the engineered *E. coli*; visual aid explaining the CarS ORF fix; breakdown of ether lipid precursors in engineered *E. coli* strains, ratios of bacterial, archaeal, and hybrid cardiolipin species as well as growth curves of EL+ strain treated with cerulenin; ion counts, list of Gro-APCL masses found in the *E. coli* mixed membrane lipidome; plasmids strains and primers; source and Genbank accession numbers of the enzymes used for ether lipid as well as isoprenoid production in the engineered *E. coli* (PDF)

## Abbreviations

PG: phosphatidylglycerol
PE: phosphatidylethanolamine
AG: archaetidylglycerol
AE: archaetidylethanolamine
EL: ether lipid
IPP: isopentenyl pyrophosphate
DMAPP: dimethylallyl pyrophosphate
Cl: cardiolipin
Gro-DPCL: glycerol-diphosphatidyl-cardiolipin
Gro-DACL: glycerol-diarchaetidyl-cardiolipin
Gro-APCL: glycerol-archaetidyl-phosphatidyl-cardiolipin
