# Supplementary material for: Optimizing Archaeal Lipid Biosynthesis in Escherichia coli
Source: ACS Synth Biol. 2024 Aug 3;13(8):2470–9. doi: 10.1021/acssynbio.4c00235 (PMC11334171; doi:10.1021/acssynbio.4c00235)
Supplement: Supplementary file 1 — sb4c00235_si_001.pdf [file sb4c00235_si_001.pdf]

Supporting information for

Optimizing Archaeal lipid biosynthesis in *Escherichia coli*

Mirthe Hoekzema, Jiayi Jiang and Arnold J. M. Driessen*

Department of Molecular Microbiology, Groningen Biomolecular Sciences and Biotechnology Institute, University of Groningen, Groningen, Netherlands

*Correspondence: a.j.m.driessen@rug.nl

Table of contents

Figure S1: *carS* open reading frames (ORF) fix.
Figure S2: Breakdown of ether lipid precursors in engineered *E. coli* strains.
Figure S3: Ratios of bacterial, archaeal and hybrid cardiolipin species.
Figure S4: Growth curves of EL+ strain treated with Cerulenin.

Table S1: Corrected ion count Archaeal lipids and their precursors (Relates to figure 1B)
Table S2: Ion counts PG species (relates to figure 1)
Table S3: Ion counts PG species (relates to figure 1)
Table S4: Corrected ion count Archaeal lipids and their precursors (Relates to figure 2)
Table S5: Ion counts PG species (relates to figure 2)
Table S6: Ion counts PE species (relates to figure 2)
Table S7: List of Gro-APCL masses found in the *E. coli* mixed membrane lipidome
Table S8: Bacterial strains used in this study
Table S9: Plasmids used in the study
Table S10: Primer sequences used in this study
Table S11: Enzymes used for ether lipid production in *E. coli*
Table S12: Enzymes used for isoprenoid overexpression

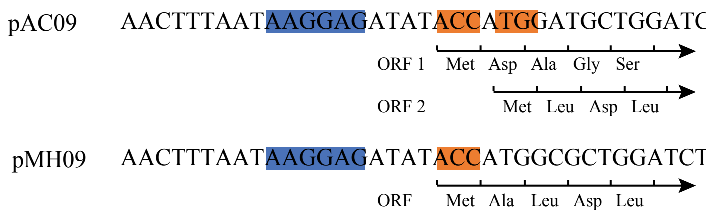

Figure S1: *carS* open reading frames (ORF) fix. The pAC09 vector from Caforio et al. 2018 has two start codons (orange box) close after the RBS (blue box) resulting in two open reading frames (arrow), one of which is out of frame. This issue is fixed in pMH09 and in all derivatives of pMH09.

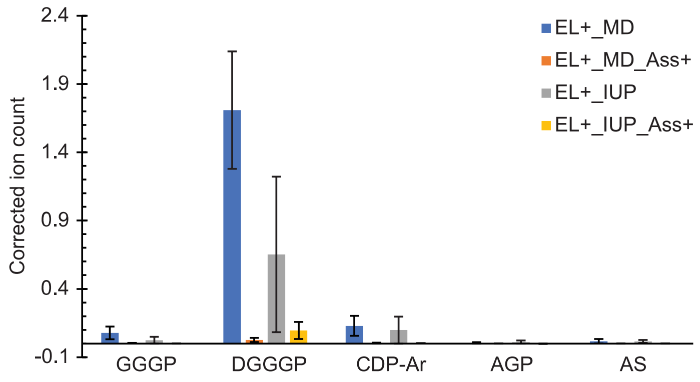

Figure S2: Breakdown of ether lipid precursors in engineered *E. coli* strains. Archaeal lipid species in strains with chromosomally integrated MEP/DOXP operon (MD) or Isoprenoid utilization pathway (IUP) were analyzed by LC-MS, normalized for the internal standard DDM, and plotted. Bars represent the mean of the datapoints (MD samples N=5 IUP samples N=3). Ass+ indicated heterologous overexpression of *M. maripaludis* archaetidylserine synthase. In case of IUP samples isoprenol was added to the media.

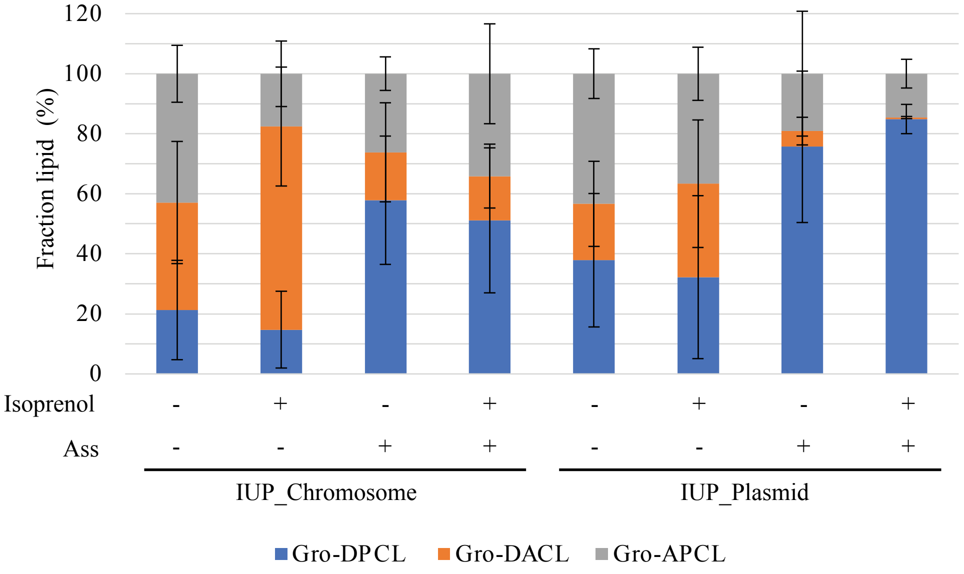

Figure S3: Ratios of bacterial, archaeal and hybrid cardiolipin species. LC-MS analysis of lipid extracts from strains with the archaeal lipid biosynthetic pathway with and without overexpression of IDI from a plasmid grown for 16 hours in M9 with 10μM IPTG in the presence and absence of 25mM isoprenol. Figure shows the ratio's between Gro_DPCL, Gro-DACL and Gro_APCL lipid species, where 100% is the total ion count of all cardiolipin species quantified. Data reflect averages ± SD (N=3).

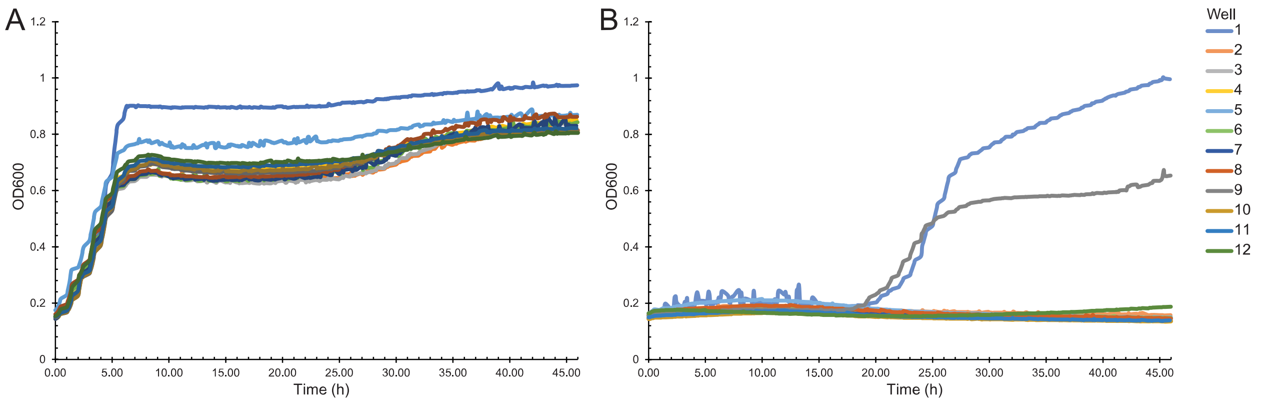

Figure S4: Growth curves of EL+ strain treated with Cerulenin. Twelve overnight cultures of the EL+ strain were diluted 1:100 in M9 media (A) and M9 + 100 µg/ml Cerulenin (B), then incubated in a plate reader at 37C with shaking and OD600 was measured every 10 minutes for 45 hours. Two out of twelve show signs of growth after approximately 20 hours.

Table S1: Corrected ion count Archaeal lipids and their precursors (Relates to figure 1B)
LC-MS analysis of lipid extracts from strains without (EL-) and with (EL+) archaeal ether lipid biosynthesis pathway, including PssA overexpression (EL+_PssA+) and Ass overexpression (EL+_Ass+) grown for 16 hours in LB with 10µM IPTG for induction of EL biosynthesis genes. Numbers given are the determined ion counts of indicated archaeal lipid species as well as precursors, divided by the ion count of the internal control (DDM).

	**EL+ a**	**EL+ b**	**EL+ c**	**EL+ d**	**EL+ e**	**EL+_PssA+ a**	**EL+_PssA+ b**	**EL+_PssA+ c**	**EL+_PssA+ d**	**EL+_PssA+ e**	**EL+_Ass+ a**	**EL+_Ass+ b**	**EL+_Ass+ c**	**EL+_Ass+ d**	**EL+_Ass+ e**
**GGGP**	0.09	0.05	0.00	0.12	0.12	ND	0.00	0.00	0.00	0.01	ND	0.00	0.00	0.01	0.00
**DGGGP**	1.22	1.28	2.40	1.83	1.83	0.10	0.02	0.02	0.05	0.05	0.05	0.02	0.01	0.04	0.02
**CDP-Ar**	0.02	0.12	0.08	0.21	0.21	0.00	0.00	0.00	0.00	0.00	0.01	0.00	0.00	0.00	0.00
**AGP**	0.00	0.01	0.01	0.00	0.00	0.00	0.00	0.00	NF	NF	0.00	0.00	0.00	NF	NF
**AG**	2.65	6.04	10.45	13.48	13.48	0.79	0.41	0.28	1.03	0.98	0.32	0.15	0.75	0.26	0.09
**AS**	0.05	0.01	0.01	0.01	0.01	0.00	0.00	0.00	0.00	0.00	0.00	0.00	0.00	0.00	0.00
**AE**	1.67	1.45	2.27	4.62	4.62	2.48	3.26	3.14	13.92	10.43	3.24	4.54	3.24	12.36	4.12

Table S2: Ion counts PG species (relates to figure 1)
LC-MS analysis of lipid extracts from strains without (EL-) and with (EL+) archaeal ether lipid biosynthesis pathway, including PssA overexpression (EL+_PssA+) and Ass overexpression (EL+_Ass+) grown for 16 hours in LB with 10µM IPTG for induction of EL biosynthesis genes. Numbers given are the determined ion counts of indicated phosphatidyl glycerol species divided by the ion count of the internal control (DDM).

	**EL+** a	**EL+** b	**EL+** c	**EL+** d	**EL+** **e**	**EL+_PssA+** a	**EL+_PssA+** b	**EL+_PssA+** c	**EL+_PssA+** d	**EL+_PssA+** **e**	**EL+_Ass+** a	**EL+_Ass+** b	**EL+_Ass+** c	**EL+_Ass+** d	**EL+_Ass+** **e**	**EL-** **a**	**EL-** **b**	**EL-** **c**
**DDM**	3.87E+07	9.18E+06	5.44E+06	6.75E+06	7.47E+06	5.13E+07	3.95E+07	5.03E+06	4.45E+06	8.91E+06	4.47E+07	1.56E+07	8.01E+06	6.92E+06	2.00E+07	3.85E+07	3.11E+07	1.01E+07
**24:0**	5.32E+02	1.18E+03	1.74E+03	NF	NF	9.88E+03	NF	2.54E+04	NF	1.23E+02	1.32E+04	1.18E+03	NF	NF	1.34E+02	2.39E+05	1.43E+05	5.50E+04
**25:0**	2.50E+03	6.05E+03	1.10E+04	NF	3.81E+02	4.46E+04	4.20E+02	6.85E+04	2.53E+03	4.13E+03	2.59E+04	4.64E+02	7.45E+03	5.07E+02	8.74E+02	2.01E+05	8.96E+04	4.25E+04
**26:0**	9.47E+04	2.52E+05	1.29E+05	3.47E+04	7.28E+04	6.10E+05	8.04E+04	4.29E+05	8.62E+04	1.46E+05	3.69E+05	4.84E+04	4.58E+04	7.58E+04	7.98E+04	3.15E+06	1.15E+06	5.71E+05
**27:0**	5.37E+04	8.57E+04	7.35E+04	7.36E+03	2.83E+04	5.30E+05	7.09E+04	2.65E+05	7.65E+04	1.02E+05	4.00E+05	4.28E+04	5.35E+04	3.42E+04	5.31E+04	2.20E+06	8.50E+05	1.74E+05
**27:1**	3.48E+03	1.40E+04	1.64E+04	1.34E+03	2.33E+03	6.55E+04	1.42E+03	1.80E+05	8.97E+03	1.72E+04	2.41E+04	5.92E+02	2.49E+04	2.18E+03	4.73E+03	1.39E+06	4.78E+05	2.26E+05
**28:0**	6.73E+05	9.34E+05	4.56E+05	2.47E+05	4.30E+05	2.67E+06	8.43E+05	1.39E+06	4.68E+05	8.92E+05	2.63E+06	7.55E+05	4.98E+05	6.01E+05	6.41E+05	1.98E+07	7.53E+06	2.28E+06
**28:1**	3.28E+05	3.06E+05	1.67E+05	8.16E+04	1.94E+05	1.14E+06	3.24E+05	7.26E+05	1.94E+05	3.78E+05	5.49E+05	2.23E+05	9.47E+04	2.10E+05	2.09E+05	7.18E+06	2.85E+06	8.20E+05
**29:0**	2.22E+05	4.75E+05	3.85E+05	4.21E+04	1.75E+05	2.51E+06	4.74E+05	1.36E+06	3.72E+05	5.86E+05	2.82E+06	4.32E+05	8.55E+05	2.34E+05	3.47E+05	1.86E+07	8.02E+06	1.81E+06
**29:1**	9.19E+04	9.16E+04	8.02E+04	5.35E+04	4.56E+04	7.16E+05	1.54E+05	3.86E+05	1.11E+05	2.04E+05	4.89E+05	7.61E+04	1.51E+05	6.04E+04	8.94E+04	5.75E+06	2.07E+06	5.11E+05
**30:0**	2.67E+06	8.88E+06	5.05E+06	2.14E+06	4.58E+06	1.90E+07	9.20E+06	1.18E+07	4.75E+06	1.06E+07	1.74E+07	1.06E+07	7.57E+06	6.51E+06	6.82E+06	1.26E+08	7.01E+07	3.99E+07
**30:1**	1.96E+06	1.91E+06	9.12E+05	6.42E+05	1.12E+06	7.75E+06	2.50E+06	2.09E+06	1.28E+06	2.30E+06	6.54E+06	2.09E+06	1.40E+06	1.58E+06	1.53E+06	2.45E+07	1.05E+07	2.48E+06
**30:2**	1.17E+05	7.16E+04	3.76E+04	3.40E+04	7.94E+04	2.95E+05	9.30E+04	7.98E+04	4.03E+04	8.73E+04	2.19E+05	1.01E+05	6.72E+03	7.90E+04	8.10E+04	7.71E+05	3.01E+05	7.27E+04
**31:0**	7.59E+05	2.13E+06	1.63E+06	2.11E+05	6.56E+05	9.43E+06	2.57E+06	3.05E+06	1.82E+06	3.02E+06	1.37E+07	3.77E+06	1.61E+06	1.50E+06	1.93E+06	3.11E+07	1.41E+07	4.85E+06
**31:1**	1.38E+06	1.39E+06	1.35E+06	5.86E+05	1.01E+06	1.29E+07	2.76E+06	5.09E+06	3.02E+06	4.63E+06	9.45E+06	1.85E+06	3.34E+06	1.51E+06	2.25E+06	4.10E+07	1.64E+07	4.61E+06
**31:2**	1.14E+04	2.20E+04	1.05E+04	1.48E+04	1.33E+04	1.43E+05	2.63E+04	7.70E+04	2.74E+04	7.20E+04	1.02E+05	1.41E+04	1.76E+04	4.00E+04	3.80E+04	1.73E+06	5.92E+05	1.45E+05
**32:0**	4.80E+06	1.47E+07	7.31E+06	4.88E+06	6.28E+06	1.73E+07	1.26E+07	8.25E+06	8.95E+06	1.99E+07	2.57E+07	2.22E+07	5.48E+06	2.07E+07	2.12E+07	3.81E+07	2.03E+07	1.54E+07
**32:1**	4.11E+07	4.61E+07	2.43E+07	1.76E+07	3.00E+07	1.18E+08	6.51E+07	4.33E+07	3.79E+07	8.01E+07	9.17E+07	5.61E+07	2.84E+07	4.55E+07	5.06E+07	1.36E+08	8.71E+07	3.72E+07
**32:2**	1.03E+06	8.52E+05	4.37E+05	3.65E+05	6.45E+05	3.61E+06	9.76E+05	4.94E+05	5.24E+05	1.04E+06	2.56E+06	8.46E+05	2.30E+05	6.58E+05	6.75E+05	5.85E+06	2.31E+06	5.56E+05
**33:0**	4.92E+05	2.27E+06	1.58E+06	2.36E+05	4.67E+05	7.63E+06	3.39E+06	9.39E+05	1.75E+06	3.36E+06	1.12E+07	6.23E+06	6.66E+05	2.32E+06	2.85E+06	3.86E+06	1.21E+06	6.58E+05
**33:1**	2.28E+07	2.01E+07	1.41E+07	1.65E+07	9.33E+06	9.97E+07	4.54E+07	1.03E+08	2.03E+07	4.03E+07	8.49E+07	3.65E+07	3.23E+07	1.94E+07	2.47E+07	3.24E+08	2.12E+08	1.58E+08
**33:2**	2.51E+06	4.37E+05	2.80E+05	2.99E+05	3.45E+05	3.58E+06	2.47E+06	9.91E+05	6.57E+05	1.10E+06	2.14E+06	2.47E+06	4.05E+05	5.61E+05	6.74E+05	1.05E+07	4.16E+06	1.36E+06
**34:0**	2.12E+05	2.63E+06	1.72E+06	8.87E+05	7.42E+05	7.47E+06	2.40E+06	7.13E+05	1.76E+06	4.10E+06	9.13E+06	4.59E+06	5.38E+05	5.38E+06	5.25E+06	1.84E+06	8.32E+05	6.42E+05
**34:1**	6.78E+07	5.93E+07	4.11E+07	3.42E+07	3.78E+07	1.70E+08	9.79E+07	4.71E+07	5.24E+07	1.08E+08	1.23E+08	9.62E+07	3.22E+07	7.36E+07	8.43E+07	1.75E+08	1.20E+08	6.20E+07
**34:2**	4.38E+06	3.84E+06	2.58E+06	2.07E+06	3.14E+06	2.02E+07	7.40E+06	1.91E+07	3.74E+06	8.46E+06	1.06E+07	4.29E+06	2.30E+06	3.61E+06	4.54E+06	7.86E+07	4.91E+07	2.43E+07
**35:0**	4.22E+04	3.10E+05	2.47E+05	2.12E+04	2.86E+04	7.78E+05	1.11E+06	6.81E+04	1.72E+05	3.33E+05	8.39E+05	1.05E+06	3.71E+04	3.46E+05	4.01E+05	1.37E+05	2.47E+04	1.79E+04
**35:1**	6.56E+06	1.01E+07	8.80E+06	4.23E+06	3.77E+06	4.31E+07	2.57E+07	6.24E+06	1.19E+07	2.19E+07	2.95E+07	2.54E+07	8.22E+06	9.76E+06	1.45E+07	5.93E+07	3.08E+07	1.23E+07
**35:2**	1.54E+06	1.05E+06	7.55E+05	9.79E+05	8.13E+05	7.17E+06	4.69E+06	1.14E+07	1.47E+06	3.07E+06	5.23E+06	2.96E+06	2.56E+06	1.36E+06	2.06E+06	7.94E+07	5.35E+07	2.81E+07

**36:0**	NF	1.04E+05	7.50E+04	6.04E+03	1.29E+04	3.36E+05	1.31E+04	1.34E+04	5.61E+04	1.29E+05	2.98E+05	3.60E+04	8.43E+03	1.87E+05	1.72E+05	4.33E+04	NF	5.99E+03
**36:1**	1.56E+06	2.06E+06	1.75E+06	1.33E+06	1.12E+06	1.79E+07	8.38E+06	1.56E+06	1.95E+06	4.83E+06	1.18E+07	8.47E+06	8.12E+05	4.10E+06	4.77E+06	1.15E+07	8.43E+06	2.65E+06
**36:2**	5.99E+06	5.16E+06	4.37E+06	2.99E+06	4.36E+06	2.36E+07	1.44E+07	1.20E+07	6.56E+06	1.32E+07	1.14E+07	6.52E+06	3.62E+06	5.73E+06	7.86E+06	6.82E+07	4.50E+07	2.01E+07
**37:1**	3.42E+04	1.04E+05	7.78E+04	3.41E+04	8.26E+03	4.52E+05	4.09E+05	3.33E+04	4.17E+04	1.13E+05	4.29E+05	5.21E+05	5.59E+04	8.54E+04	1.39E+05	2.83E+05	8.27E+04	4.84E+04
**37:2**	8.54E+04	3.27E+04	2.10E+05	5.48E+02	4.46E+04	7.77E+05	2.88E+05	2.86E+05	7.63E+04	1.42E+05	6.68E+05	2.37E+05	1.06E+06	1.01E+05	1.62E+05	4.54E+06	2.18E+06	9.04E+05
**38:2**	NF	1.89E+04	3.89E+04	1.25E+04	2.77E+04	2.71E+05	8.00E+04	9.43E+04	5.18E+04	7.98E+04	1.86E+05	2.97E+02	6.11E+04	6.16E+04	7.44E+04	6.76E+05	5.70E+05	1.69E+05

Table S3: : Ion counts PE species (relates to figure 1)
LC-MS analysis of lipid extracts from strains without (EL-) and with (EL+) archaeal ether lipid biosynthesis pathway, including PssA overexpression (EL+_PssA+) and Ass overexpression (EL+_Ass+) grown for 16 hours in LB with 10µM IPTG for induction of EL biosynthesis genes. Numbers given are the determined ion counts of indicated phosphatidyl ethanolamine species divided by the ion count of the internal control (DDM).

	**EL+** a	**EL+** b	**EL+** c	**EL+** d	**EL+** **e**	**EL+_PssA+** a	**EL+_PssA+** b	**EL+_PssA+** c	**EL+_PssA+** d	**EL+_PssA+** **e**	**EL+_Ass+** a	**EL+_Ass+** b	**EL+_Ass+** c	**EL+_Ass+** d	**EL+_Ass+** **e**	**EL-** **a**	**EL-** **b**	**EL-** **c**
**DDM**	3.87E+07	9.18E+06	5.44E+06	6.75E+06	7.47E+06	5.13E+07	3.95E+07	5.03E+06	4.45E+06	8.91E+06	4.47E+07	1.56E+07	8.01E+06	6.92E+06	2.00E+07	3.85E+07	3.11E+07	1.01E+07
**24:0**	4.94E+05	1.71E+05	7.45E+04	1.62E+04	6.96E+04	2.81E+05	1.45E+05	2.68E+05	2.62E+04	3.79E+04	1.72E+05	1.89E+05	7.99E+03	5.00E+04	5.10E+04	6.18E+06	4.86E+06	7.71E+05
**24:1**	3.07E+05	2.77E+05	1.82E+05	1.49E+04	9.56E+04	3.78E+05	9.40E+04	2.25E+05	3.84E+04	5.51E+04	1.80E+05	8.09E+04	7.34E+03	4.87E+04	4.47E+04	2.00E+06	1.27E+06	5.41E+05
**25:0**	1.04E+05	6.71E+04	4.28E+04	1.22E+03	1.96E+04	1.90E+05	2.73E+04	7.14E+04	1.41E+04	1.65E+04	1.13E+05	2.36E+04	1.01E+04	1.24E+04	1.71E+04	8.73E+05	5.26E+05	8.22E+04
**25:1**	2.76E+05	1.38E+05	8.37E+04	2.10E+04	4.64E+04	2.55E+05	1.06E+05	6.50E+05	2.18E+04	3.59E+04	1.52E+05	1.18E+05	2.09E+04	2.92E+04	3.45E+04	1.41E+07	8.96E+06	1.76E+06
**26:0**	2.10E+06	1.26E+06	5.09E+05	2.50E+05	5.88E+05	2.24E+06	5.41E+05	3.63E+05	1.84E+05	3.18E+05	1.49E+06	8.27E+05	1.57E+05	4.59E+05	4.67E+05	5.86E+06	2.89E+06	8.28E+05
**26:1**	1.28E+06	5.60E+05	2.50E+05	3.44E+04	2.52E+05	7.25E+05	3.70E+05	2.97E+05	9.62E+04	1.43E+05	3.07E+05	3.77E+05	1.91E+04	1.59E+05	1.46E+05	7.17E+06	3.40E+06	6.16E+05
**27:0**	6.84E+05	5.24E+05	2.91E+05	6.94E+04	2.44E+05	1.23E+06	1.86E+05	2.29E+05	1.72E+05	2.22E+05	1.38E+06	2.95E+05	1.17E+05	1.89E+05	2.46E+05	9.19E+05	7.97E+05	2.17E+05
**27:1**	4.31E+05	2.30E+05	1.38E+05	1.04E+04	9.53E+04	6.14E+05	1.18E+05	2.07E+05	6.97E+04	9.16E+04	2.58E+05	1.18E+05	2.25E+04	5.68E+04	7.37E+04	5.85E+06	3.06E+06	8.21E+05
**28:0**	1.08E+07	6.47E+06	2.82E+06	3.37E+06	4.30E+06	1.20E+07	4.52E+06	1.83E+06	1.54E+06	3.41E+06	1.09E+07	7.76E+06	2.19E+06	3.76E+06	4.10E+06	1.28E+07	4.72E+06	4.36E+06
**28:1**	7.32E+06	3.85E+06	1.43E+06	4.51E+05	2.54E+06	5.89E+06	1.43E+06	4.35E+05	8.42E+05	1.31E+06	3.35E+06	2.18E+06	2.29E+05	2.03E+06	1.72E+06	4.56E+06	2.64E+06	7.32E+05
**28:2**	2.70E+05	1.19E+05	3.75E+04	2.82E+03	7.83E+04	2.10E+05	6.10E+04	5.13E+03	1.91E+04	2.50E+04	7.40E+04	5.15E+04	8.33E+02	5.31E+04	3.99E+04	1.60E+05	8.66E+04	1.57E+04
**29:0**	3.57E+06	1.86E+06	1.46E+06	7.27E+05	1.20E+06	7.48E+06	2.07E+06	1.78E+06	8.69E+05	1.48E+06	7.75E+06	3.21E+06	2.15E+06	1.09E+06	1.46E+06	2.58E+07	1.01E+07	3.73E+06
**29:1**	2.25E+06	1.19E+06	6.80E+05	2.15E+05	6.97E+05	2.79E+06	7.55E+05	6.98E+05	4.05E+05	5.50E+05	2.08E+06	9.29E+05	3.85E+05	6.51E+05	6.39E+05	8.57E+06	7.21E+06	1.80E+06
**29:2**	7.56E+04	2.96E+04	1.51E+04	1.34E+02	1.42E+04	1.08E+05	1.97E+04	2.67E+04	3.06E+03	5.04E+03	5.23E+04	2.10E+04	4.58E+03	1.29E+04	1.39E+04	4.81E+05	2.65E+05	8.99E+04
**30:0**	2.52E+07	1.81E+07	1.15E+07	2.76E+07	1.86E+07	3.56E+07	1.51E+07	1.96E+07	8.15E+06	2.10E+07	2.98E+07	2.82E+07	2.08E+07	1.70E+07	2.13E+07	1.36E+08	8.61E+07	7.23E+07
**30:1**	5.94E+07	3.36E+07	1.59E+07	1.10E+07	2.31E+07	3.69E+07	1.97E+07	3.72E+06	7.29E+06	1.23E+07	3.17E+07	2.98E+07	6.68E+06	1.65E+07	1.41E+07	1.40E+07	1.47E+07	5.15E+06
**30:2**	3.22E+06	1.72E+06	7.11E+05	1.87E+05	2.05E+06	2.18E+06	4.77E+05	7.54E+04	3.04E+05	4.21E+05	1.50E+06	1.10E+06	6.56E+04	1.48E+06	1.02E+06	3.63E+05	3.06E+05	1.25E+05
**31:0**	8.47E+06	4.36E+06	4.55E+06	2.81E+06	3.12E+06	3.13E+07	9.84E+06	5.06E+06	2.58E+06	6.34E+06	3.10E+07	1.41E+07	4.45E+06	4.02E+06	5.38E+06	5.52E+07	1.89E+07	1.14E+07
**31:1**	3.91E+07	1.75E+07	1.36E+07	1.07E+07	1.22E+07	4.66E+07	1.69E+07	6.04E+06	9.41E+06	1.54E+07	3.88E+07	2.28E+07	9.30E+06	9.64E+06	1.08E+07	4.50E+07	3.25E+07	1.03E+07
**31:2**	2.02E+06	6.14E+05	3.16E+05	1.67E+05	6.00E+05	1.10E+06	2.84E+05	1.75E+05	1.41E+05	2.16E+05	8.76E+05	5.94E+05	9.62E+04	6.15E+05	4.28E+05	1.02E+06	9.44E+05	5.16E+05
**32:0**	1.22E+07	7.45E+06	2.09E+02	2.79E+07	9.04E+06	3.70E+07	1.83E+07	1.22E+03	7.69E+06	2.17E+07	3.64E+07	2.42E+07	1.09E+03	1.40E+07	2.05E+07	4.16E+07	1.92E+07	1.19E+03
**32:1**	2.40E+08	1.58E+08	1.27E+08	1.38E+08	1.59E+08	2.07E+08	1.47E+08	4.92E+07	9.15E+07	1.35E+08	1.86E+08	1.83E+08	7.10E+07	1.31E+08	1.27E+08	9.75E+07	9.79E+07	3.62E+07
**32:2**	2.65E+07	1.27E+07	7.33E+06	2.42E+06	1.23E+07	1.35E+07	7.18E+06	9.35E+05	3.07E+06	6.17E+06	1.02E+07	1.06E+07	1.25E+06	7.40E+06	6.04E+06	3.57E+06	2.73E+06	9.44E+05
**33:0**	4.28E+06	2.09E+06	2.74E+06	2.19E+06	1.47E+06	2.03E+07	7.39E+06	1.50E+06	1.85E+06	5.19E+06	1.70E+07	1.08E+07	8.28E+05	3.66E+06	4.80E+06	1.59E+07	1.73E+06	8.58E+05
**33:1**	2.10E+08	1.31E+08	1.01E+08	1.62E+08	1.11E+08	2.56E+08	1.53E+08	1.21E+08	6.93E+07	1.23E+08	1.97E+08	2.02E+08	1.13E+08	1.07E+08	1.09E+08	2.85E+08	2.57E+08	1.76E+08
**33:2**	2.11E+07	7.02E+06	5.14E+06	4.27E+06	6.68E+06	1.54E+07	7.29E+06	1.77E+06	2.61E+06	4.96E+06	1.06E+07	9.95E+06	2.50E+06	5.06E+06	4.72E+06	1.11E+07	8.72E+06	2.59E+06

**34:0**	4.66E+06	9.53E+05	1.48E+06	5.13E+06	9.66E+05	2.16E+07	6.53E+06	8.42E+05	1.14E+06	5.13E+06	1.17E+07	9.40E+06	4.68E+05	2.52E+06	4.25E+06	8.12E+06	1.28E+06	5.36E+05
**34:1**	1.93E+08	1.27E+08	9.61E+07	1.33E+08	1.32E+08	2.08E+08	1.44E+08	5.53E+07	8.20E+07	1.32E+08	1.48E+08	1.76E+08	6.72E+07	1.18E+08	1.16E+08	1.52E+08	1.50E+08	7.60E+07
**34:2**	2.84E+07	1.57E+07	1.69E+07	1.20E+07	1.96E+07	2.41E+07	1.80E+07	2.23E+07	1.19E+07	1.77E+07	1.63E+07	8.26E+06	1.36E+07	1.44E+07	1.42E+07	6.41E+07	6.04E+07	3.01E+07
**35:0**	4.84E+05	1.26E+05	2.35E+05	1.76E+05	8.01E+04	6.33E+06	1.32E+06	1.24E+05	1.57E+05	5.25E+05	2.15E+06	2.21E+06	3.89E+04	4.06E+05	5.63E+05	4.65E+05	6.80E+04	2.56E+04
**35:1**	7.22E+07	2.70E+07	2.15E+07	4.10E+07	2.21E+07	1.27E+08	6.75E+07	7.61E+06	1.83E+07	3.94E+07	7.27E+07	9.57E+07	3.51E+07	3.08E+07	3.26E+07	8.72E+07	5.46E+07	1.53E+07
**35:2**	1.49E+07	7.66E+06	6.86E+06	4.87E+06	8.65E+06	1.55E+07	1.23E+07	1.53E+07	5.17E+06	9.53E+06	8.70E+06	4.69E+06	1.31E+07	7.58E+06	7.91E+06	3.93E+07	3.73E+07	2.96E+07
**36:0**	1.27E+05	2.38E+04	5.69E+04	1.33E+05	1.86E+04	2.43E+06	4.36E+05	2.83E+04	3.88E+04	2.07E+05	6.95E+05	7.35E+05	8.97E+03	1.03E+05	2.01E+05	3.33E+05	3.82E+04	1.28E+04
**36:1**	3.83E+07	5.81E+06	3.81E+06	7.64E+06	6.26E+06	4.40E+07	2.05E+07	1.29E+06	3.26E+06	9.41E+06	1.75E+07	3.13E+07	1.89E+06	7.50E+06	7.55E+06	2.46E+07	9.11E+06	2.09E+06
**36:2**	1.79E+07	1.16E+07	1.24E+07	7.17E+06	1.51E+07	1.55E+07	1.49E+07	1.02E+07	1.02E+07	1.41E+07	8.80E+06	8.22E+06	1.34E+07	1.03E+07	1.04E+07	4.43E+07	4.36E+07	2.01E+07
**37:1**	8.64E+05	1.47E+05	1.26E+05	3.21E+05	1.29E+05	4.10E+06	1.47E+06	4.70E+04	1.79E+05	4.20E+05	1.33E+06	3.10E+06	2.16E+05	5.13E+05	4.81E+05	1.58E+06	3.97E+05	6.54E+04
**37:2**	2.79E+06	1.12E+06	6.45E+05	1.47E+06	6.60E+05	4.70E+06	2.24E+06	3.35E+05	4.39E+05	1.07E+06	2.31E+06	2.83E+06	6.01E+06	1.17E+06	1.09E+06	6.74E+06	4.62E+06	1.18E+06
**38:0**	2.36E+05	3.40E+04	2.48E+04	2.01E+04	2.92E+04	7.46E+05	3.74E+05	8.57E+03	2.02E+04	8.22E+04	4.04E+05	1.14E+06	4.67E+03	1.01E+05	9.81E+04	4.31E+05	8.68E+04	1.65E+04
**38:2**	5.80E+05	1.04E+05	7.28E+04	8.59E+04	1.03E+05	8.49E+05	6.45E+05	4.90E+04	6.48E+04	1.74E+05	4.21E+05	1.19E+06	3.49E+05	1.58E+05	1.53E+05	1.80E+06	7.42E+05	1.26E+05
**39:2**	4.14E+03	NF	NF	NF	NF	2.61E+04	1.95E+04	NF	NF	1.26E+02	1.73E+04	9.14E+04	NF	4.03E+02	6.70E+02	2.67E+04	7.47E+03	NF

Table S4: Corrected ion count Archaeal lipids and their precursors (Relates to figure 2)

Ion counts from indicated archaeal lipid species divided by an internal control as determined by LC-MS analysis of lipid extracts. Strains all have the archaeal lipid biosynthetic pathway. When Ass is overexpressed this is indicated by Ass+. C indicates chromosomal expression of the IUP (ScCK, AtIPK, EcIDI) while P indicates plasmid based expression. Alternatively only IDI (IDI) or IUP version 2 (SmDAGK, MvIPK, EcIDI) are expressed from a plasmid (IDI_V2). All strains are grown for 16 hours in M9 with 10µM IPTG for induction of EL biosynthesis genes, when indicated 25mM isoprenol was added to the media (Iso).

		GGGP	DGGGP	CDP-Ar	AGP	AG	AS	AE
**EL+_IUP_C**	**a**	0.03	0.01	0.00	0.00	11.96	NF	0.11
	**b**	0.00	0.02	0.00	0.01	11.47	NF	0.04
	**c**	0.00	0.01	0.00	0.00	4.29	NF	0.03
**EL+_IUP_C_Iso**	**a**	0.06	0.50	0.06	0.01	21.43	0.02	1.01
	**b**	0.01	1.41	0.23	0.03	16.71	0.02	1.99
	**c**	0.00	0.05	0.00	0.00	6.20	0.00	0.05
**EL+_Ass+_C**	**a**	0.00	0.01	0.00	0.00	1.72	0.00	3.96
	**b**	NF	0.01	0.00	NF	1.11	0.00	3.19
	**c**	0.00	0.01	0.00	NF	0.84	0.00	2.34
**EL+_Ass+_C_Iso**	**a**	0.00	0.17	0.00	0.00	2.90	0.00	4.99
	**b**	0.00	0.02	0.00	NF	1.34	0.00	2.76
	**c**	0.00	0.10	0.00	0.00	1.79	0.00	3.29
**EL+_IUP_P**	**a**	0.02	0.01	0.00	0.01	8.35	NF	0.08
	**b**	0.00	0.00	0.00	0.00	5.18	NF	0.09
	**c**	0.00	0.02	0.00	0.00	4.71	NF	0.05
**EL+_IUP_P_Iso**	**a**	0.05	1.08	0.07	0.15	20.08	0.00	0.49
	**b**	0.00	0.19	0.02	0.00	7.59	0.00	0.49
	**c**	0.01	0.73	0.08	0.05	8.12	0.01	0.29
**EL+_Ass+_P**	**a**	0.00	0.00	0.00	0.00	0.40	0.00	1.29
	**b**	0.00	0.02	0.00	0.00	0.60	0.00	2.44
	**c**	0.00	0.02	0.00	0.00	1.05	0.00	2.86
**EL+_Ass+_P_Iso**	**a**	0.00	0.50	0.77	0.01	3.81	0.00	3.52
	**b**	0.00	0.38	0.03	0.01	2.30	0.00	3.15
	**c**	0.00	0.41	0.02	0.01	2.15	0.00	3.24
**IDI-only**	**a**	0.01	0.01	0.00	0.00	5.75	NF	0.04
	**b**	0.00	0.01	0.00	0.00	1.68	NF	0.01
	**c**	0.00	0.03	0.01	0.00	3.92	0.00	0.04
**IDI-only_Iso**	**a**	0.01	0.01	NF	0.01	5.46	NF	0.03
	**b**	0.00	0.01	0.00	0.01	2.23	0.00	0.00
	**c**	0.00	0.07	0.00	0.00	4.77	0.00	0.04
**EL+_IUP-V2_Iso**	**a**	0.00	0.36	0.25	0.01	5.24	0.00	0.41
	**b**	0.00	0.66	0.02	0.02	8.18	0.04	0.60
	**c**	0.01	1.17	0.02	0.03	10.58	0.03	0.52
**EL+_Ass+_IUP-V2_Iso**	**a**	0.00	0.19	0.17	0.01	2.55	0.00	3.83
	**b**	0.00	0.31	0.02	0.01	2.31	0.00	3.55
	**c**	0.00	0.71	0.00	0.00	4.02	0.01	5.11

Table S5: Ion counts PG species (relates to figure 2)

Raw ion counts from indicated PG species as determined by LC-MS analysis of lipid extracts. Strains all have the archaeal lipid biosynthetic pathway. When Ass is overexpressed this is indicated by Ass+. C indicates chromosomal expression of the IUP (ScCK, AtIPK, EcIDI) while P indicates plasmid based expression. Alternatively only IDI (IDI) or IUP version 2 (SmDAGK, MvIPK, EcIDI) are expressed from a plasmid (IDI_V2). All strains are grown for 16 hours in M9 with 10µM IPTG for induction of EL biosynthesis genes, when indicated 25mM isoprenol was added to the media (Iso).

		DDM	30:0	31:0	31:1	32:0	32:1	33:1	34:1	34:2	35:1	35:2	36:1	36:2
**EL+_IUP_C**	**a**	9.12E+06	5.67E+07	2.49E+05	6.41E+06	4.06E+07	1.62E+07	1.76E+08	2.31E+07	4.77E+06	1.58E+08	2.80E+06	1.98E+06	3.55E+07
	**b**	8.01E+06	2.41E+07	8.14E+04	2.64E+06	1.10E+07	5.92E+06	7.90E+07	8.96E+06	2.09E+06	7.99E+07	1.32E+06	7.26E+05	1.52E+07
	**c**	4.00E+07	3.82E+07	5.01E+04	4.57E+06	2.59E+07	1.73E+07	1.70E+08	3.98E+07	4.83E+06	1.74E+08	2.93E+07	2.96E+06	3.82E+07
**EL+_IUP_C_Iso**	**a**	1.14E+07	1.03E+07	2.18E+04	1.33E+06	1.18E+07	4.31E+06	7.90E+07	9.34E+06	7.44E+05	1.04E+08	1.10E+06	1.13E+06	6.46E+06
	**b**	8.71E+06	2.19E+06	9.29E+03	1.46E+05	3.06E+06	9.04E+05	1.31E+07	2.44E+06	2.02E+05	2.35E+07	3.65E+05	2.47E+05	1.46E+06
	**c**	3.90E+07	1.66E+07	2.26E+04	1.70E+06	1.57E+07	1.41E+07	1.18E+08	2.72E+07	2.13E+06	1.43E+08	1.34E+07	2.05E+06	1.30E+07
**EL+_Ass+_C**	**a**	5.70E+06	2.16E+07	1.13E+05	3.59E+06	1.66E+07	4.87E+06	9.73E+07	9.49E+06	2.13E+06	8.52E+07	1.14E+06	1.01E+06	1.77E+07
	**b**	2.32E+07	5.58E+07	1.46E+05	9.66E+06	3.72E+07	1.96E+07	2.16E+08	3.85E+07	6.35E+06	1.88E+08	4.27E+07	3.69E+06	6.97E+06
	**c**	3.79E+07	6.71E+07	1.45E+05	1.31E+07	4.53E+07	2.47E+07	2.46E+08	5.15E+07	5.43E+06	2.18E+08	5.68E+07	5.01E+06	6.73E+07
**EL+_Ass+_C_Iso**	**a**	1.36E+07	3.58E+07	1.84E+05	3.92E+06	3.93E+07	1.04E+07	1.51E+08	2.53E+07	1.88E+06	1.55E+08	2.16E+06	3.00E+06	2.43E+07
	**b**	3.28E+07	2.41E+07	4.46E+04	1.41E+06	2.09E+07	1.40E+07	1.10E+08	2.17E+07	1.59E+06	1.46E+08	8.26E+06	2.62E+06	9.63E+06
	**c**	3.62E+07	6.86E+07	1.63E+05	7.83E+06	5.73E+07	2.33E+07	2.15E+08	3.95E+07	3.15E+06	2.32E+08	2.99E+07	4.07E+06	3.28E+07
**EL+_IUP_P**	**a**	1.09E+07	7.14E+07	6.09E+04	8.60E+06	3.56E+07	1.37E+07	2.14E+08	1.61E+07	5.46E+06	1.64E+08	3.19E+06	1.51E+06	3.88E+07
	**b**	2.66E+07	2.07E+07	2.21E+04	1.25E+06	1.29E+07	4.13E+06	1.30E+08	8.76E+06	2.56E+06	1.24E+08	1.74E+06	1.07E+06	1.77E+07
	**c**	3.22E+07	4.47E+07	1.22E+05	9.70E+06	5.08E+07	3.42E+07	1.97E+08	5.65E+07	1.04E+07	1.79E+08	8.35E+06	5.35E+06	6.75E+07
**EL+_IUP_P_Iso**	**a**	1.03E+07	4.97E+07	8.22E+04	3.54E+06	5.71E+07	4.17E+07	1.85E+08	6.84E+07	4.51E+06	1.70E+08	8.69E+06	3.62E+06	1.79E+07
	**b**	2.71E+07	7.89E+06	3.31E+04	4.94E+05	9.08E+06	5.94E+06	7.36E+07	1.37E+07	1.25E+06	8.50E+07	1.65E+06	1.08E+06	5.50E+06
	**c**	2.68E+07	1.54E+07	9.47E+04	2.22E+06	4.15E+07	4.71E+07	1.36E+08	7.30E+07	6.48E+06	1.35E+08	1.02E+07	5.57E+06	2.81E+07
**EL+_Ass+_P**	**a**	4.20E+07	3.79E+07	3.06E+04	3.35E+06	3.02E+07	1.19E+07	2.41E+08	1.71E+07	7.16E+06	2.02E+08	3.48E+06	1.47E+06	4.02E+07
	**b**	4.07E+07	7.92E+07	9.72E+04	5.69E+06	4.89E+07	6.72E+07	2.60E+08	1.22E+08	1.65E+07	2.41E+08	4.93E+07	1.05E+07	8.04E+07
	**c**	3.44E+07	9.32E+07	1.59E+05	1.89E+07	6.71E+07	4.92E+07	2.52E+08	6.49E+07	1.42E+07	2.17E+08	1.04E+07	6.88E+06	8.52E+07
**EL+_Ass+_P_Iso**	**a**	1.76E+07	1.58E+07	1.54E+04	7.17E+05	2.43E+07	9.54E+06	1.34E+08	2.69E+07	1.94E+06	1.34E+08	2.99E+06	1.51E+06	8.87E+06
	**b**	3.44E+07	7.06E+07	6.86E+04	3.13E+06	5.94E+07	2.90E+07	1.90E+08	6.06E+07	3.73E+06	1.97E+08	2.54E+07	4.71E+06	2.50E+07
	**c**	2.70E+07	4.51E+07	2.90E+05	4.08E+06	5.01E+07	2.25E+07	1.92E+08	5.14E+07	5.61E+06	1.61E+08	4.24E+07	4.76E+06	4.24E+07
**No-IUP**	**a**	1.59E+07	7.16E+07	2.00E+05	1.67E+07	5.14E+07	1.76E+07	2.19E+08	2.82E+07	1.09E+07	1.75E+08	3.70E+06	3.57E+06	7.10E+07
	**b**	8.23E+06	2.74E+07	6.31E+04	5.27E+06	1.51E+07	5.01E+06	1.16E+08	8.14E+06	3.23E+06	7.67E+07	1.44E+06	8.06E+05	2.39E+07
	**c**	1.29E+07	4.20E+07	7.32E+04	9.42E+06	2.35E+07	8.62E+06	1.53E+08	1.40E+07	6.04E+06	9.70E+07	1.79E+06	1.35E+06	4.23E+07
**IDI-only**	**a**	1.33E+07	7.63E+07	8.20E+04	1.22E+07	3.97E+07	1.70E+07	2.33E+08	1.65E+07	6.39E+06	1.74E+08	3.55E+06	1.44E+06	3.80E+07

	**b**	3.42E+07	8.02E+07	1.35E+05	2.49E+07	7.55E+07	2.29E+07	2.94E+08	2.97E+07	1.39E+07	2.32E+08	6.06E+06	3.98E+06	8.69E+07
	**c**	3.21E+07	3.35E+07	1.17E+05	6.91E+06	3.57E+07	7.68E+07	2.02E+08	1.29E+08	4.10E+07	1.91E+08	4.68E+07	1.83E+07	1.27E+08
**IDI-only_Iso**	**a**	1.70E+07	9.63E+07	8.46E+04	1.17E+07	5.94E+07	3.63E+07	2.46E+08	4.05E+07	5.58E+06	2.20E+08	7.00E+06	2.70E+06	3.24E+07
	**b**	1.86E+07	4.80E+07	5.84E+04	7.58E+06	5.06E+07	4.02E+07	1.76E+08	5.09E+07	5.16E+06	1.62E+08	8.81E+06	3.39E+06	3.29E+07
	**c**	2.93E+07	1.90E+07	1.11E+05	2.15E+06	2.18E+07	1.01E+08	1.53E+08	1.76E+08	3.95E+07	1.39E+08	3.09E+07	2.45E+07	9.35E+07
**EL+_IUP-V2_Iso**	**a**	2.03E+07	1.35E+07	9.86E+03	5.44E+05	1.74E+07	5.97E+06	1.08E+08	1.79E+07	1.31E+06	1.07E+08	2.20E+06	1.07E+06	7.03E+06
	**b**	2.07E+07	7.12E+06	2.20E+04	8.95E+05	1.33E+07	5.08E+06	9.03E+07	1.57E+07	1.30E+06	8.37E+07	9.86E+06	1.02E+06	9.86E+06
	**c**	2.10E+07	1.15E+07	2.71E+04	1.91E+06	2.42E+07	1.37E+07	1.35E+08	3.59E+07	3.06E+06	1.22E+08	5.25E+06	2.25E+06	1.98E+07
**EL+_Ass+_IUP-V2_Iso**	**a**	3.07E+07	1.90E+07	2.22E+04	7.21E+05	2.60E+07	1.32E+07	1.37E+08	4.85E+07	1.76E+06	1.47E+08	3.95E+06	3.92E+06	1.01E+07
	**b**	3.03E+07	4.17E+07	9.09E+04	4.65E+06	5.19E+07	1.51E+07	1.96E+08	3.99E+07	3.54E+06	1.63E+08	3.90E+07	4.20E+06	3.90E+07
	**c**	2.22E+07	3.68E+07	6.78E+04	6.25E+06	4.18E+07	1.98E+07	2.09E+08	4.67E+07	4.81E+06	1.84E+08	6.04E+06	5.68E+06	4.59E+07

Table S6: Ion counts PE species (relates to figure 2)

Raw ion counts from indicated PE species as determined by LC-MS analysis of lipid extracts. Strains all have the archaeal lipid biosynthetic pathway. When Ass is overexpressed this is indicated by Ass+. C indicates chromosomal expression of the IUP (ScCK, AtIPK, EcIDI) while P indicates plasmid based expression. Alternatively only IDI (IDI) or IUP version 2 (SmDAGK, MvIPK, EcIDI) are expressed from a plasmid (IDI_V2). All strains are grown for 16 hours in M9 with 10µM IPTG for induction of EL biosynthesis genes, when indicated 25mM isoprenol was added to the media (Iso).

		30:0	30:1	31:0	31:1	32:0	32:1	33:1	34:1	34:2	35:1	35:2	36:2
**EL+_IUP_C**	**a**	1.02E+08	5.16E+06	3.38E+05	1.52E+07	5.99E+07	1.36E+07	2.43E+08	1.26E+07	2.31E+07	1.74E+08	2.28E+06	1.07E+08
	**b**	7.37E+07	2.49E+06	5.81E+05	1.24E+07	4.76E+07	7.62E+06	1.88E+08	9.22E+06	1.32E+07	1.39E+08	2.23E+06	8.12E+07
	**c**	1.28E+08	9.45E+06	4.01E+05	2.82E+07	6.90E+07	2.63E+07	3.08E+08	3.62E+07	3.39E+07	2.34E+08	5.23E+06	1.57E+08
**EL+_IUP_C_Iso**	**a**	8.42E+07	1.48E+06	3.05E+05	9.14E+06	6.25E+07	1.79E+07	2.28E+08	3.14E+07	1.00E+07	2.00E+08	3.09E+06	8.19E+07
	**b**	6.68E+07	9.68E+05	6.87E+05	7.38E+06	5.68E+07	1.17E+07	1.82E+08	2.99E+07	6.48E+06	1.58E+08	3.01E+06	5.60E+07
	**c**	6.30E+07	2.77E+06	1.49E+05	1.47E+07	2.40E+07	3.00E+07	2.81E+08	6.31E+07	1.83E+07	2.20E+08	7.25E+06	1.16E+08
**EL+_Ass+_C**	**a**	4.88E+07	2.50E+06	4.50E+05	1.16E+07	4.05E+07	8.78E+06	1.64E+08	1.23E+07	1.13E+07	1.16E+08	1.84E+06	6.37E+07
	**b**	8.16E+07	6.97E+06	4.56E+05	2.73E+07	4.75E+07	2.99E+07	2.87E+08	3.20E+07	3.36E+07	1.99E+08	4.01E+06	1.39E+08
	**c**	7.41E+07	7.84E+06	4.58E+05	2.99E+07	3.92E+07	3.58E+07	3.08E+08	4.21E+07	3.99E+07	2.11E+08	4.94E+06	1.56E+08
**EL+_Ass+_C_Iso**	**a**	5.44E+07	1.85E+06	8.87E+05	1.20E+07	7.50E+07	2.24E+07	2.16E+08	3.98E+07	1.09E+07	1.81E+08	3.58E+06	7.50E+07
	**b**	4.69E+07	1.87E+06	2.92E+05	6.68E+06	5.85E+07	1.66E+07	2.09E+08	5.47E+07	8.57E+06	1.96E+08	6.80E+06	6.55E+07
	**c**	4.23E+07	2.99E+06	2.84E+05	1.41E+07	2.92E+07	3.11E+07	2.76E+08	5.58E+07	1.75E+07	2.19E+08	5.13E+06	1.12E+08
**EL+_IUP_P**	**a**	9.67E+07	5.53E+06	2.14E+05	1.68E+07	4.94E+07	1.49E+07	2.50E+08	1.65E+07	2.24E+07	1.61E+08	2.66E+06	9.98E+07
	**b**	8.00E+07	5.80E+06	1.41E+05	1.45E+07	2.09E+07	2.08E+07	2.50E+08	2.75E+07	1.92E+07	1.77E+08	4.42E+06	1.21E+08
	**c**	1.34E+08	9.90E+06	5.25E+05	2.95E+07	7.88E+07	4.10E+07	3.10E+08	4.88E+07	4.11E+07	2.26E+08	7.07E+06	1.53E+08
**EL+_IUP_P_Iso**	**a**	1.02E+08	3.61E+06	4.12E+05	1.27E+07	6.70E+07	3.58E+07	2.57E+08	6.25E+07	1.38E+07	2.01E+08	8.62E+06	7.14E+07
	**b**	4.75E+07	3.06E+06	1.05E+05	1.04E+07	1.26E+07	2.59E+07	2.28E+08	5.23E+07	1.25E+07	1.90E+08	7.72E+06	9.30E+07
	**c**	8.86E+07	4.61E+06	4.19E+05	1.81E+07	5.00E+07	5.32E+07	2.69E+08	8.99E+07	2.65E+07	2.18E+08	1.20E+07	1.05E+08
**EL+_Ass+_P**	**a**	8.02E+07	1.12E+07	1.75E+05	1.89E+07	1.81E+07	4.40E+07	2.86E+08	3.99E+07	2.90E+07	1.82E+08	5.20E+06	1.33E+08
	**b**	7.02E+07	7.36E+06	4.76E+05	1.81E+07	2.80E+07	5.58E+07	2.83E+08	8.58E+07	3.43E+07	1.92E+08	1.25E+07	1.27E+08
	**c**	7.17E+07	1.03E+07	7.18E+05	2.52E+07	3.18E+07	4.74E+07	3.05E+08	6.18E+07	4.08E+07	2.03E+08	7.78E+06	1.50E+08
**EL+_Ass+_P_Iso**	**a**	3.78E+07	3.65E+06	6.87E+04	6.01E+06	1.09E+07	3.46E+07	2.15E+08	5.93E+07	1.12E+07	1.67E+08	7.88E+06	7.28E+07
	**b**	6.12E+07	3.02E+06	3.48E+05	1.34E+07	5.03E+07	2.98E+07	2.63E+08	7.19E+07	1.54E+07	2.13E+08	9.08E+06	8.90E+07
	**c**	1.92E+07	2.42E+06	1.80E+05	1.21E+07	6.97E+06	2.34E+07	1.82E+08	4.40E+07	1.16E+07	1.39E+08	6.78E+06	7.74E+07
**No-IUP**	**a**	1.11E+08	9.11E+06	4.06E+05	2.53E+07	6.81E+07	2.47E+07	2.63E+08	2.84E+07	3.08E+07	1.93E+08	2.84E+06	1.28E+08
	**b**	5.60E+07	3.56E+06	1.48E+05	1.39E+07	2.89E+07	8.22E+06	1.63E+08	1.13E+07	1.18E+07	1.18E+08	1.43E+06	6.62E+07
	**c**	8.63E+07	6.72E+06	1.66E+05	2.41E+07	4.55E+07	1.55E+07	2.15E+08	1.57E+07	2.30E+07	1.57E+08	1.84E+06	9.66E+07
**IDI-only**	**a**	1.26E+08	8.30E+06	3.36E+05	2.52E+07	6.51E+07	2.34E+07	2.78E+08	2.02E+07	2.88E+07	1.77E+08	3.06E+06	1.08E+08

	**b**	1.17E+08	1.03E+07	3.63E+05	2.96E+07	6.51E+07	3.16E+07	3.34E+08	2.89E+07	3.95E+07	2.13E+08	5.04E+06	1.52E+08
	**c**	1.12E+08	1.01E+07	4.83E+05	2.14E+07	5.73E+07	7.46E+07	2.82E+08	1.17E+08	5.42E+07	1.98E+08	3.00E+07	1.35E+08
**IDI-only_Iso**	**a**	1.17E+08	5.04E+06	3.57E+05	2.02E+07	6.69E+07	3.25E+07	2.92E+08	4.15E+07	2.02E+07	2.15E+08	5.62E+06	1.02E+08
	**b**	6.07E+07	3.28E+06	1.83E+05	1.59E+07	2.79E+07	2.88E+07	2.25E+08	5.50E+07	1.62E+07	1.69E+08	9.40E+06	8.34E+07
	**c**	6.15E+07	4.83E+06	2.45E+05	1.44E+07	1.56E+07	8.84E+07	2.56E+08	1.62E+08	4.47E+07	1.72E+08	2.86E+07	1.03E+08
**EL+_IUP-V2_Iso**	**a**	4.34E+07	3.25E+06	6.51E+04	6.61E+06	1.06E+07	2.86E+07	2.03E+08	5.40E+07	1.02E+07	1.55E+08	7.21E+06	7.25E+07
	**b**	2.30E+07	1.49E+06	6.36E+04	9.60E+06	6.27E+06	1.74E+07	1.64E+08	3.35E+07	9.12E+06	1.22E+08	5.61E+06	6.32E+07
	**c**	1.07E+08	4.43E+06	4.54E+05	1.93E+07	7.40E+07	4.19E+07	2.55E+08	7.36E+07	1.84E+07	2.18E+08	8.08E+06	9.85E+07
**EL+_Ass+_IUP-V2_Iso**	**a**	3.52E+07	2.36E+06	8.68E+04	4.40E+06	9.65E+06	3.41E+07	1.85E+08	7.87E+07	8.41E+06	1.58E+08	8.24E+06	5.36E+07
	**b**	2.15E+07	1.99E+06	1.67E+05	1.37E+07	1.07E+07	1.98E+07	1.88E+08	4.24E+07	1.04E+07	1.51E+08	5.90E+06	7.58E+07
	**c**	4.08E+07	3.16E+06	2.42E+05	1.52E+07	2.94E+07	3.41E+07	2.29E+08	6.26E+07	1.57E+07	1.98E+08	6.46E+06	9.90E+07

Table S7: List of Gro-APCL masses found in the *E. coli* mixed membrane lipidome
List of all the suspected Gro-APCL species found in the *E. coli* strain with the ether lipid biosynthetic pathway and IUP expression. Theoretical as well of observed masses and retention time (RT) are given. Lipid core refers to the amount of carbon units and saturations, this number is given for the varying phosphatidyl tail, as well as the phosphatidyl and the archaetidyl combined (total).

Lipid core phosphatidyl	Lipid core total	Theoretical m/z $[M-H]^-$	observed m/z $[M-H]^-$	ppm error	RT
27:0	67:0	1349.92817	1349.92712	-0.77782	27.28
28:0	68:0	1363.94382	1363.94165	-1.59097	27.78
29:0	69:0	1377.95947	1377.95667	-2.03199	28.3
30:0	70:0	1391.97512	1391.97205	-2.2055	28.89
30:1	70:1	1389.95947	1389.95801	-1.05039	27.8
31:1	71:1	1403.97512	1403.97351	-1.14674	28.57
32:0	72:0	1420.00642	1420.00305	-2.37323	29.82
32:1	72:1	1417.99077	1417.98853	-1.5797	28.82
33:1	**73:1**	1432.00642	1432.00342	-2.09496	29.55
34:1	**74:1**	1446.02207	1446.01917	-2.0055	29.74
34:2	74:2	1444.00642	1444.00537	-0.72714	29.19
35:1	**75:1**	1460.03772	1460.03381	-2.67801	30.43
35:2	75:2	1458.02207	1458.0199	-1.48832	29.43
36:2	76:2	1472.03772	1472.03516	-1.73909	30.19
37:2	77:2	1486.05337	1486.05042	-1.98512	30.35
38:2	78:2	1500.06902	1500.06604	-1.98658	31.02

Table S8 Bacterial strains used in this study

Strain	Name	Genome integration	Plasmids	Source/ Reference
JM109(DE3)	wt	none		Commercial strain
MEP/DOXP+	EL-	*idi-ispDF-dsx*	pET-Duet, pRSF-Duet	Caforio et al. 2018
MH154	EL+	*idi-ispDF-dsx*	pMS148, pMH09	This study
MH105	EL+_PssA+	*idi-ispDF-dsx*	pMS148, pMH10	This study
MH107	EL+_Ass+	*idi-ispDF-dsx*	pMS148, pMH12	This study
MH166	EL+_IUP_C	P_{pro4}_*ScCK_AtIPK_idi*	pMS148, pMH09	This study
MH169	EL+_Ass+_C	P_{pro4}_*ScCK_AtIPK_idi*	pMS148, pMH12	This study
MH167	EL+_IUP_P	none	pMS148, pMH09, pMH27	This study
MH172	EL+_Ass+_P	none	pMS148, pMH12,pMH27	This study
MH168	EL+_No_IUP	none	pMS148, pMH09, pACYC	This study
MH175	IDI_only	P_{pro4}_*idi*	pMH128. pMH09, pMH35	This study
MH173	EL+_IUP-V2	none	pMS148, pMH09, pMH42	This study
MH174	EL+_Ass+_IUP-V2	none	pMS148, pMH12, pMH42	This study

Table S9 Plasmids used in the study

Name	Description (origin; antibiotic marker; promoter; operon)	Source/ reference
pET-Duet	ColE1; $Amp^{R;}$ P_{T7lac}	Novagen
pRSF-Duet	RSF1030; Km^R; P_{T7lac}	Novagen
pACYC-Duet	P15A; Cm^R; P_{T7lac}	Novagen
pMS148	ColE1; Amp^R; P_{T7lac}_*araM; T7lac_crtE*	(Caforio et al., 2018)
pAC027	RSF1030; Km^R; P_{T7lac}_ *carS-his6*, *his6-gggps, his6-dgggps*	(Caforio et al., 2018)
pAC029	RSF1030; Km^R; P_{T7lac}_ *carS-his6*, *his6-gggps, his6-dgggps, pssA-his6*	(Caforio et al., 2018)
pMH09	RSF1030; Km^R; P_{T7lac}_*carS, his6-gggps, dgggps*	This study
pMH10	RSF1030; Km^R; P_{T7lac}_*carS, his6-gggps, dgggps*; T7*lac_pssA*	This study
pMH12	RSF1030; Km^R; P_{T7lac}_*carS, his6-gggps, dgggps*; T7*lac_ass*	This study
pSEVA228-pro4IUPi	RK2, KnR, P_{pro4}_*ck, ipk, idi*	(Chatzivasileiou et al., 2019)
pMH27	P15A; Cm^R; P_{pro4}_*ck, ipk, idi*	This study
pMH35	P15A; Cm^R; P_{pro4}_ *idi*	This study
pMH42	P15A; Cm^R; P_{pro4}_*dagk, ipk, idi*	This study

Amp^R = ampicillin resistance, Km^R = kanamycin resistance, Cm^R = chloramphenicol resistance

Table S10 – Primer sequences used in this study

Name	Sequence 5'- 3'
MHO-021	GAAAAACCATGGCGCTGGATCTGATTCTGAAAAC
MHO-022	GTTTTTGAGCTCTTATTTGAAGAACGAGCC
MHO-023	AGGATCCGAATTCATGCATC
MHO-024	ATGGATATCAAAGCCTATTTCGAGC
MHO-025	phos- AGGTCTTTACCTCTCTTATACTTAAC
MHO-026	GTTTTTGGATCCTTACCAAGGAACTTCTTTCAGG
MHO-027	GAAAAACATATGAATTACATCCCCTGTATG
MHO-028	GAAAAACGATCGTTAATTCCATCTCCCAGACTC

MHO-031	GAAAAACATATGTTCAAGATCCGCAAC
MHO-032	GAAAAACGATCGTTACATTTTCATCTTGCTGATACC
MHO-071	AGGCTACCGCCTGTTAGCGTAAACCACCACATAACTATGGTTTACAGCTAGCTCAGTCC
MHO-072	TCTTCAATTTGTTGTGTACGGGTTTTCATGTGCAGATGCTGATTCTCACCAATAAAAAACGCC
MHO-073	AGGCTACCGCCTGTTAGCGTAAACCACCACATAACTATGGTTCTAGAGCACAGCTAACAC
MHO-074	TCTTCAATTTGTTGTGTACGGGTTTTCATGTGCAGATGCTGATATAGTTCCTCCTTTCAGC
MHO-077	GTTTTTGGATCCTTCTAGAGCACAGCTAACAC
MHO-078	GTTTTTGCGGCCGCGATATAGTTCCTCCTTTCAGC
MHO-079	GTTTTTGCGGCCGCCCATTCATCCGCTTATTATCAC
MHO-080	GTTTTTGGATCCCTCACTCATTAGGCACCG
MHO-090	phos-AACTAAGGAGGTCTATATGCAAACG
MHO-091	GAAACCGTTGTGGTCTCCC
MHO-123	GGAGGTTTAAATGCCGATGGATCTGCGTG
MHO-124	TTTCTTGTACTTAGTGGAACAGCAGGAACC
MHO-125	GTTCCACTAAGTACAAGAAAAGTCAGTAGTCTAAG
MHO-126	CTCCTTAGTTTTAGTTAATGCTGGTGCCTTTAAC
MHO-127	CATTAACTAAAACTAAGGAGGTCTATATGCAAACGG
MHO-128	CCATCGGCATTTAAACCTCCTTAATGTGTTTCCTTGC

Table S11 - Enzymes used for ether lipid production in *E. coli*

Gene/ Locus	Source	Accession number (GenBank)	Enzyme	Function	Expression
araM	*Bacillus subtilis*	NP_390754.1	Glycerol-1-phosphate dehydrogenase	DHAP+NADH→G1P	plasmid
crtE	*Pantoea ananatis*, codon optimized	BAA14124.1	Geranylgeranyl diphosphate synthase	IPP+DMAPP→GGPP	plasmid
MmarC7_1004	*Methanococcus maripaludis*, codon optimized	ABR66071.1	Geranylgeranyl glyceryl phosphate synthase	G1P+GGPP→GGGP	plasmid
MmarC7_RS04845	*M. maripaludis*, codon optimized	ABR66017.1	Di-O-Geranylgeranyl glyceryl phosphate synthase	GGPP+GGGP→DGGGP	plasmid
CarS (AF1740)	*Archaeoglobus fulgidus*, codon optimized	AAB89505.1	CDP-archaeol synthase	DGGGP+CTP→CDP-archaeol	plasmid
pssA	*B. subtilis*	CAB12021.1	Phosphatidylserine synthase	CDP-archaeol→AS	plasmid
ass	*M. maripaludis*, codon optimized	CAF30727.1	Archaetidylserine synthase	CDP-archaeol→AS	plasmid
pssA	*E. coli*		Phosphatidylserine synthase	CDP-archaeol→AS	native
psd	*E. coli*		Phosphatidylserine decarboxylase	AS→AE	native
pgsA	*E. coli*		Phosphatidylglycerophosphate synthase	CDP-archaeol→AGP	native

pgpA	*E. coli*	Phosphatidylglycerophosphatase	AGP→AG	native

Table S12 - Enzymes used for isoprenoid overexpression

Gene	Source	Accession number	Enzyme	Pathway	Expression	Reference
ispD	*E. coli*	AAF43207.1	2-C-methyl-D-erythritol 4-phosphate cytidylyltransferase	MEP/DOXP	chromosome	(Caforio et al., 2018)
ispF	*E. coli*	AAA79837.1	2-C-methyl-D-erythritol 2,4-cyclodiphosphate synthase	MEP/DOXP	chromosome	(Caforio et al., 2018)
dsx	*E. coli*	AAC46162.1	1-deoxy-D-xylulose-5-phosphate synthase	MEP/DOXP	chromosome	(Caforio et al., 2018)
idi	*E. coli*	AAD26812.1	Isopentenyl-diphosphate Delta-isomerase	MEP/DOXP / IUP/IUP-V2	chromosome / plasmid	(Caforio et al., 2018; Chatzivasileiou et al., 2019)
ck	*Saccharomyces cerevisiae*, codon optimized	AAA34499.1	Choline kinase	IUP	chromosome / plasmid	(Chatzivasileiou et al., 2019)
ipk	*Arabidopsis thaliana*, codon optimized	AAN12957.1	isopentenyl phosphate kinase	IUP	chromosome / plasmid	(Chatzivasileiou et al., 2019)
dagk	*Streptococcus mutans*, condon optimized	AAA26867.1	diacylglycerol kinase	IUP-V2	plasmid	(Ma et al., 2022)
ipk	*Methanococcus vannielii*, codon optimized	WP_012065782.1	isopentenyl phosphate kinase	IUP-V2	plasmid	(Ma et al., 2022)
